# Atomic Force Microscopy Analysis of the Role of Major DNA-Binding Proteins in Organization of the Nucleoid in *Escherichia coli*

**DOI:** 10.1371/journal.pone.0072954

**Published:** 2013-08-12

**Authors:** Ryosuke L. Ohniwa, Hiroki Muchaku, Shinji Saito, Chieko Wada, Kazuya Morikawa

**Affiliations:** 1 Division of Biomedical Science, Faculty of Medicine, University of Tsukuba, Tennoh-dai, Tsukuba, Ibaraki, Japan; 2 Yoshida Biological Laboratory, Yamashina-ku, Kyoto, Kyoto, Japan; University of Oklahoma, United States of America

## Abstract

Bacterial genomic DNA is packed within the nucleoid of the cell along with various proteins and RNAs. We previously showed that the nucleoid in log phase cells consist of fibrous structures with diameters ranging from 30 to 80 nm, and that these structures, upon RNase A treatment, are converted into homogeneous thinner fibers with diameter of 10 nm. In this study, we investigated the role of major DNA-binding proteins in nucleoid organization by analyzing the nucleoid of mutant *Escherichia coli* strains lacking HU, IHF, H–NS, StpA, Fis, or Hfq using atomic force microscopy. Deletion of particular DNA-binding protein genes altered the nucleoid structure in different ways, but did not release the naked DNA even after the treatment with RNase A. This suggests that major DNA-binding proteins are involved in the formation of higher order structure once 10-nm fiber structure is built up from naked DNA.

## Introduction

Both prokaryotic and eukaryotic cells store large size genomic DNA with various proteins and RNAs. Bacterial genomic DNA is localized within the cell in a region known as the “nucleoid” [1]. In eukaryotic cells, genomic DNA exists in the form of chromatin and is packed into a well-defined nucleus [2]. In both cases, a set of distinct structural DNA-binding proteins (e.g., histones in eukaryotic cells) plays a major role in organizing DNA into higher-order structures. In the case of eukaryotic chromatin, particular proteins (i.e., histones and condensins) mediate the step-wise folding of genomic DNA into higher-order architectures such as the nucleosome, 30-nm chromatin, 300-nm chromatin, and metaphase chromosomes. In contrast, how bacterial DNA-binding proteins participate in organizing genomic DNA within the cell remains largely unknown.

Several DNA-binding proteins are believed to act as major structural proteins in the *Escherichia coli* nucleoid, including heat-unstable nucleoid protein (HU), integration host factor protein (IHF), histone-like nucleoid structuring protein (H–NS), suppressor of td- phenotype A (StpA), factor for inversion stimulation (Fis), and host factor for phage RNA Qβ replication (Hfq) [1]. Various *in vitro* reconstitution studies have elucidated the properties of these proteins, such as their sequence preferences and how they affect DNA looping, DNA bending, and DNA zippering [3–12], but it is still unclear how these proteins contribute to nucleoid organization *in vivo*.

We have applied an “on-substrate lysis procedure” to analyze the architecture of the nucleoid using atomic force microscopy (AFM) [13–15]. Analysis of log phase *E. coli* cells by AFM revealed that fibrous structures with widths between 30 nm and 80 nm are released upon lysis. In stationary phase cells a tightly-packed structure was observed upon lysis instead of the fibrous structures. In both log and stationary phases, treatment of lysed cells with RNase A resulted in disruption of the fibrous or tightly-packed structures into thinner fibers dominantly with width of 10 nm [16]. These structures are common in bacteria such as *Staphylococcus aureus* and *Clostridium perfringens* [16,17].

The aim of the present study was to determine how the major DNA-binding proteins associated with the 10-nm or thicker fibrous structure found in actively dividing bacteria influence nucleoid architecture. We utilized AFM to analyze the nucleoid of mutant strains lacking the major DNA-binding proteins HU, IHF, H–NS, StpA, Fis, and Hfq.

## Results

### Fibrous structures in log phase *Escherichia coli* cells

**Figure 1:**
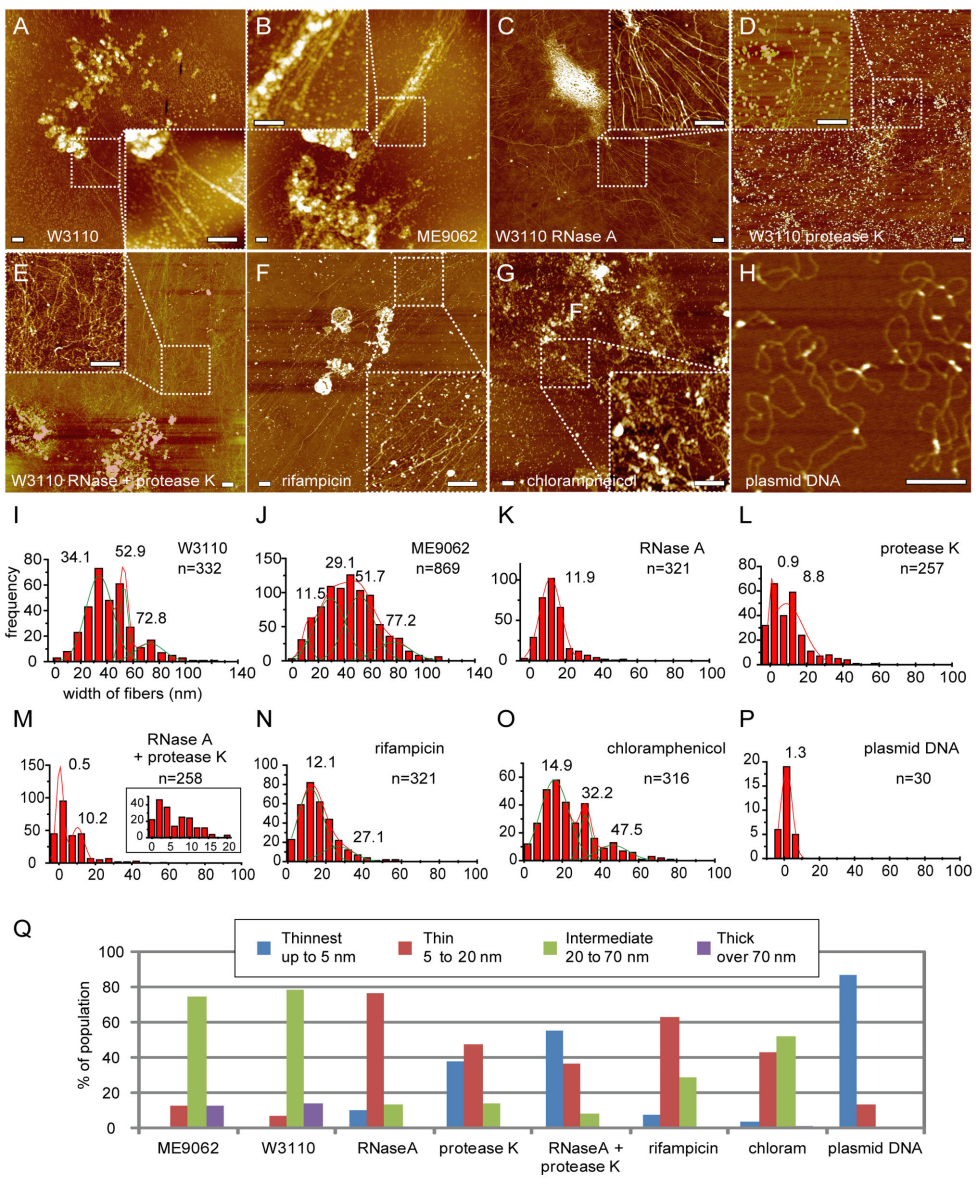
Lysed *E*. ***coli* cells treated with RNase A and protease K**. Lysed log phase *E*. *coli* W3110 (A, I) and ME9062 (B, J) cells. Lysed log phase W3110 cells were treated with RNase A (C, K), protease K (D, L), or RNase A subsequent to protease K (E, M). Log phase *E*. *coli* W3110 cells were treated in culture with 100 μg/mL of rifampicin (F, N) or 100 μg/mL of chloramphenicol (G, O) for 60 min, and were lysed. (A–G) AFM images and (I–O) graphs showing the population distribution of the width of released fibers. An inset in (M) shows the population distribution with smaller bin size under 20 nm. (H) AFM image of naked plasmid DNA (pRSFDuet-1, 3829 bp, Novagen) and (P) graph showing the width of DNA. Solid lines were obtained by Gaussian fitting, and the estimated peaks are as follows: (I) 34.1 ± 8.5, 52.9 ± 2.7, and 72.8 ± 9.2 nm (mean ± SD, n = 332 total observations), (J) 11.5 ± 3.1, 29.1 ± 9.8, 51.7 ± 9.4, and 77.2 ± 11.2 nm (n = 869), (K) 11.9 ± 7.3 nm (n = 321), (L) 0.9 ± 0.9 and 8.8 ± 7.9 nm (n = 257), (M) 0.5 ± 1.6 and 10.2 ± 3.0 nm (n = 258), (N) 12.1 ± 5.7 nm (mean ± SD) and 27.1 ± 7.0 nm (n = 321 total observations), (O) 14.9 ± 6.4, 32.2 ± 2.5 nm, and 47.5 ± 7.1 nm (n = 316), and (P) 1.3 ± 2.7 nm (n = 30). (Q) Fibers were classified into 4 categories according to their widths. Fibers with width up to 5 nm were classified as “Thinnest”, fibers with width of 5 to 20 nm were classified as “Thin”, fibers with width of 20 nm to 70 nm were classified as “Intermediate”, and fibers with width greater than 70 nm were classified as “Thick”. At least 6 nucleoids derived from at least 2 separate experiments were analyzed (Table S1). Scale bars in the AFM images represent 500 nm.

When we lysed log phase *E. coli* cells on cover-glass and examined them using AFM, we observed fibrous structures around the cell debris (Figure 1A, 1B, Figure S1A, S1B). We arbitrarily selected several area showing fibrous structures, and then magnified the selected areas at a scale of 2 μm x 2 μm to analyze the fibrous structures in greater detail. We drew 8 x 8 lines at the regular intervals with 250 nm on the images and measured the width of the fibers at the intersecting points (“grid-analysis”; see Materials and Methods & Figure S2) in order to evaluate the population distribution of fiber widths. Next, we applied a “circular cone model” to estimate the real dimensions of fiber width from the apparent dimensions determined from the AFM images (See Materials and Methods & Figure S3) [16]. This step was required to eliminate the “tip-effect” derived from the edge curvature and tilt angle of the AFM cantilever. Using this approach, we estimated that the width of the naked plasmid DNA (2 nm in the B-form) was 1.3 ± 2.7 nm (Figure 1H, 1P, Figure S4D). We applied the “grid-analysis” and “circular cone model” to all the cases examined in this study. The maximum width of the fibers was about 130 nm in both the wild type (W3110) strain and the parental strain of the deletion mutants (ME9062) (Figure 1I, 1J, Table 1). When the fiber thicknesses were classified into 4 categories, “Thinnest” (up to 5 nm), “Thin” (5 to 20 nm), “Intermediate” (20 to 70 nm) and “Thick” (over 70 nm), over 70% of the fibers were categorized in “Intermediate” (Figure 1Q). We also re-evaluated the population distribution of RNase A- and rifampicin-treated nucleoids, which we have demonstrated that the majority of fiber widths shifted to about 10 nm [16]. Consistent with the previous results, over 60% of the fiber structures were classified into "Thin" category (Figure 1C, 1F, 1K, 1N, 1Q, Figure S1C). The treatment with chloramphenicol, which inhibits the translation, also increased the population in "Thin" category (Figure 1G, 1O, 1Q, Figure S4B, S4C).

**Table 1:** Escherichia coli strains used in this study.

Strains	Genotype	Parent strain	Source
W3110	Prototroph		
ME9062 (BW25113)	*rrnB3*, Δ*lacZ4787*, *hsdR514*, Δ(*araBAD*)*567*, Δ(*rhaBAD*)*568*, *rph-1*		KEIO collection [23]
YK1100	*trpC*9941	W3110	Wada et al[27]
Δ*hupA*	Δ*hupA* + the same as ME9062	ME9062	KEIO collection [23]
Δ*hupB*	Δ*hupB* + the same as ME9062	ME9062	KEIO collection [23]
Δ*himA*	Δ*himA* + the same as ME9062	ME9062	KEIO collection [23]
Δ*himD*	Δ*himD* + the same as ME9062	ME9062	KEIO collection [23]
YK1304	Δ*hupA/ΔhupB*	YK1100	Wada et al[27]
YK2741	Δ*hupA/*Δ*hupB/*Δ*himA*	YK1100	Kano and Imamoto [28]
Δ*hns*	Δ*hns* + the same as ME9062	ME9062	KEIO collection [23]
Δ*stpA*	Δ*stpA* + the same as ME9062	ME9062	KEIO collection [23]
Δ*fis*	Δ*fis* + the same as ME9062	ME9062	KEIO collection [23]
Δ*hfq*	Δ*hfq* + the same as ME9062	ME9062	KEIO collection [23]

### Treating lysed cells with protease K exposes naked DNA

Treating lysed *E. coli* cells with protease K resulted in a dramatic change in the population distribution of fiber widths (Figure 1D, 1L, Figure S1D, S5C, S5G). The maximum fiber width was reduced to less than 60 nm, and the majority of the fibers was shifted into “Thinnest” and “Thin” (Figure 1L, 1Q). In the histogram, two peaks around 10 nm and 1 nm appeared. Additional treatment with RNase A did not diminish the 1-nm peak nor decrease the population of “Thinnest”, in which about 90% of naked DNA population was classified (Figure 1E, 1M, 1Q, Figure S4A). Treatment with RNase A alone resulted in slight exposure of ”Thinnest” fibers, and in a shift in the predominant fiber width to ”Thin” category (Figure 1C, 1K, Figure S1C, S5D, S5H) [16]. These results indicate that proteins are involved in formation of the fiber structures (>10 nm) from naked DNA.

### The lack of a major DNA-binding protein alters the population distribution of fiber widths

**Figure 2:**
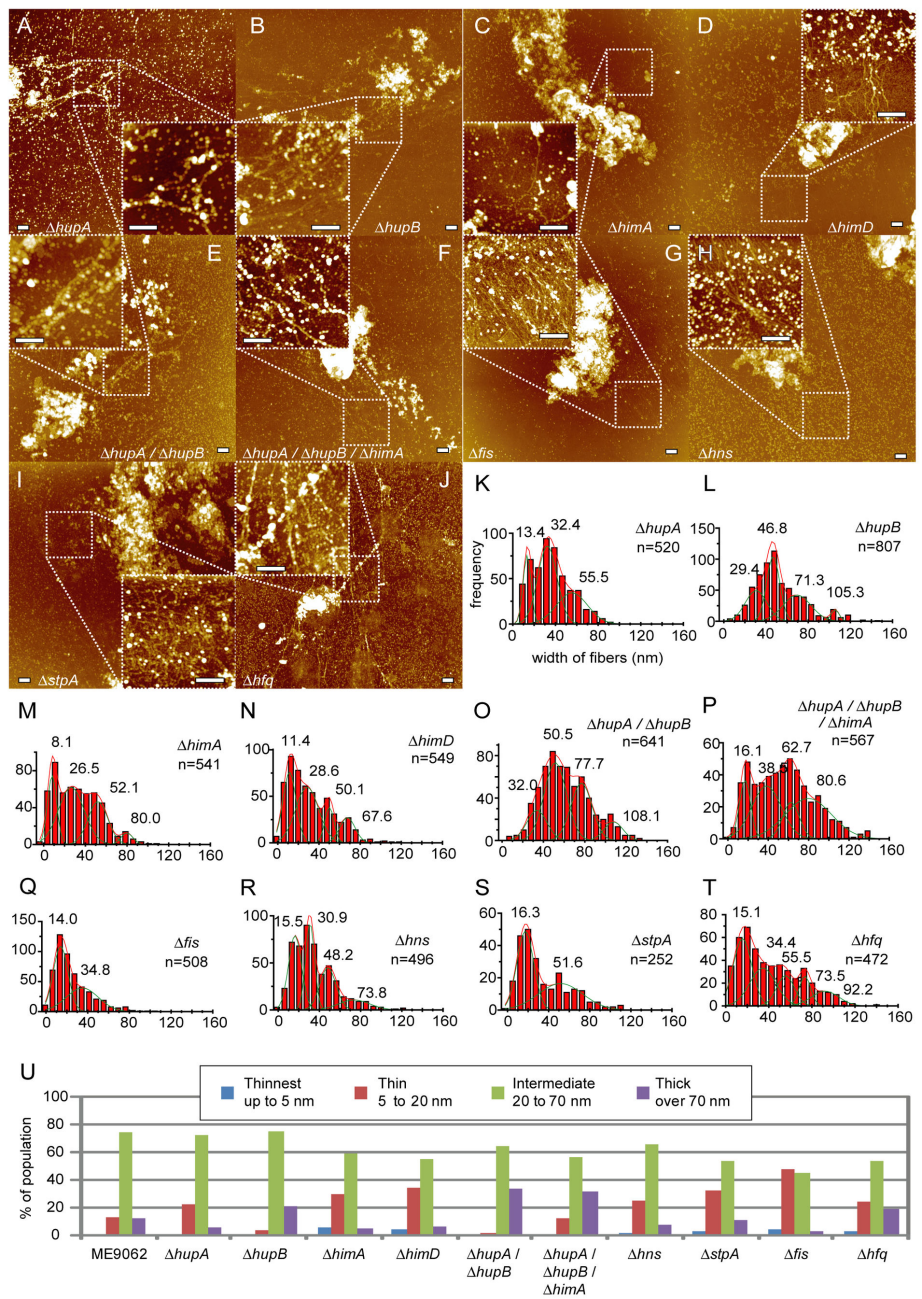
Analysis of the nucleoid of deletion mutants. AFM images of lysed deletion mutant cells (A–J). Population distribution of fiber widths (K–T). Solid lines were obtained by Gaussian fitting, and the estimated peaks are as follows: (K) 13.4 ± 3.2 nm (mean ± SD), 32.4 ± 7.7 nm, and 55.5 ± 12.3 nm (n = 520 total observations), (L) 29.4 ± 8.6, 46.8 ± 5.9, 71.3 ± 10.3, and 105.3 ± 3.4 nm (n = 807), (M) 8.1 ± 3.8, 26.5 ± 10.1, 52.1 ± 8.0, and 80.0 ± 4.9 nm (n = 541), (N) 11.4 ± 5.2, 28.6 ± 8.5, 50.1 ± 3.8, and 67.6 ± 6.3 nm (n = 549), (O) 32.0 ± 8.4, 50.5 ± 9.2, 77.7 ± 8.8, and 108.1 ± 9.7 nm (n = 641), (P) 16.1 ± 4.9, 38.5 ± 11.3, 62.7 ± 6.8, and 80.6 ± 20.0 nm (n = 567), (Q) 14.0 ± 6.1 and 34.8 ± 13.1 nm (n = 508), (R) 15.5 ± 5.4 nm, 30.9 ± 3.7 nm, 48.2 ± 6.7, and 73.8 ± 12.9 nm (n = 496), (S) 16.3 ± 6.9 and 51.6 ± 18.3 nm (n = 252), and (T) 15.1 ± 7.5, 34.4 ± 10.3, 55.5 ± 7.0, 73.5 ± 4.6, and 92.2 ± 9.9 nm (n = 472). (U) Categorization of fibers into 4 categories, “Thinnest”, “Thin”, “Intermediate” and “Thick”, as described in Figure 1. At least 8 nucleoids derived from at least 2 separate experiments were analyzed (Table S1). Scale bars in the AFM images represent 500 nm.

**Figure 3:**
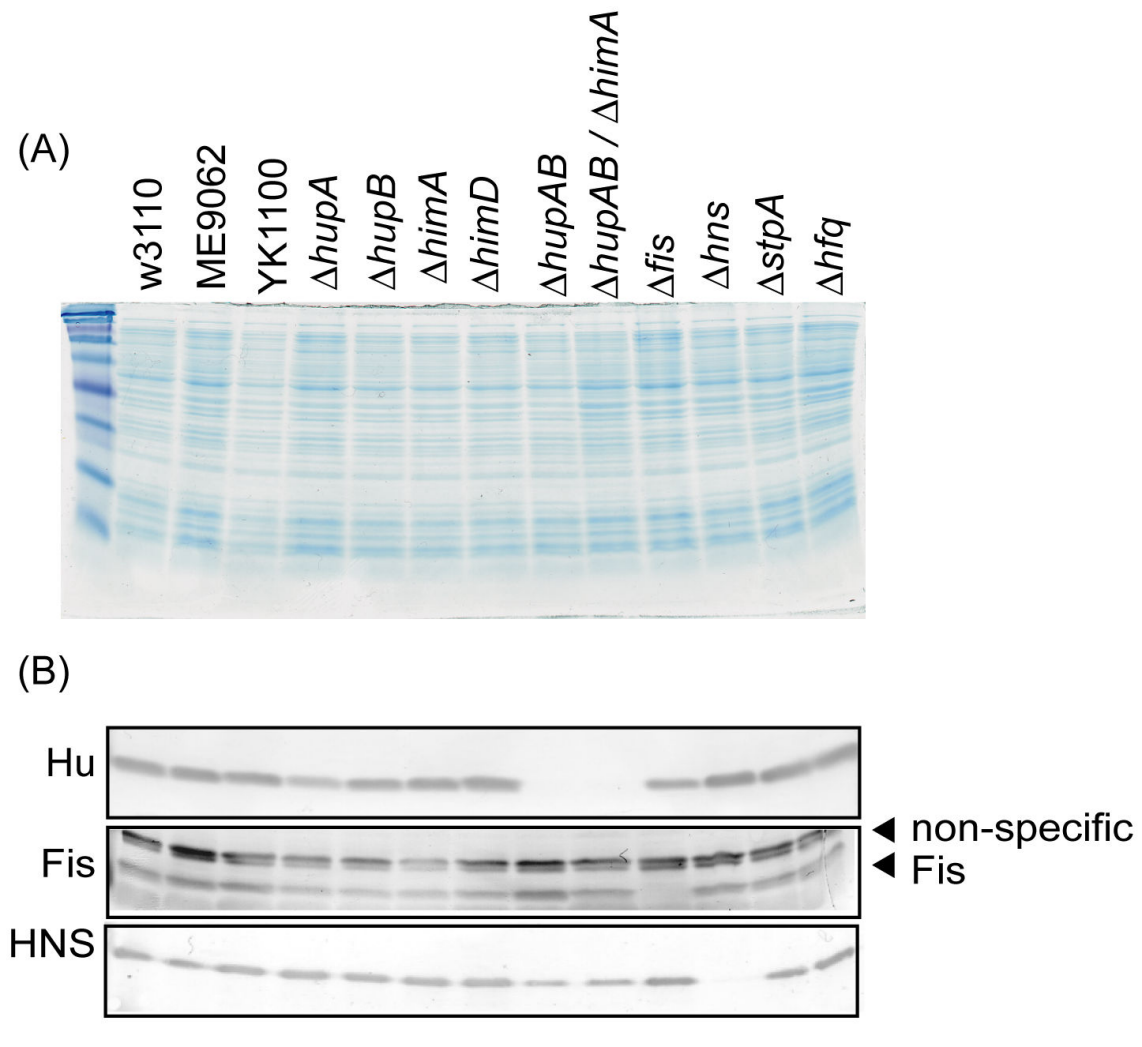
Analysis of DNA-binding protein levels in the deletion mutants. (A) SDS-PAGE of deletion mutant whole-cell lysates. The gel was stained with Coomassie Brilliant Blue. (B) Western blots against HU, Fis, and H–NS. The primary antibodies were rabbit polyclonal antibodies [20] and the secondary antibody was alkaline phosphatase-conjugated anti-rabbit IgG. The signals were detected using Western Blue (Promega, USA).

To determine whether major DNA-binding proteins are involved in formation of the nucleoid structure, we examined the nucleoid released from DNA-binding protein deletion mutants (Table 1). Heat-unstable nucleoid protein HU is expressed by most bacterial species, and is one of the most prominent proteins specific to the nucleoid [18]. The HU protein can function as either a homo- or heterodimer composed of HUα and/or HUβ subunits, which are encoded by the genes *hupA* and *hupB*, respectively. Deletion of *hupA* resulted in a decrease in the population of “Thick” fibers and an increase in the population of ”Thin” fibers (Figure 2A, 2K, 2U, Figure S6A). In contrast, deletion of *hupB* resulted in a decrease in the population of ”Thin” fibers and an increase in the population of “Thick” fibers (Figure 2B, 2L, 2U, Figure S6B). Western blot analysis showed that the lack of *hupB* did not change the amount of Huα, and the lack of *hupA* slightly decreased the amount of Huβ in our experimental condition (Figure 3). In Δ*hupA*/Δ*hupB* double knockout cells, the populations of ”Thick” fibers increased in comparison to Δ*hupB* cells (Figure 2E, 2O, 2U, Figure S7A).

Integration host factor protein is a homologue of HU, and is also a dimer, composed of IHF-A and IHF-B subunits encoded by the *himA* and *himD* genes, respectively. In contrast to HU mutants, deletion of either *himA* or *himD* resulted in a increase in the population of ”Thin” fibers (Figures 2C, 2D, 2M, 2N, Figure S6C, S6D). The lack of *himA* and *himD* did not change the total amount of Hu in cells (Figure 3). In case of the Δ*hupA/*Δ*hupB/*Δ*himA* triple knockout mutant, the population of ”Thick” fibers increased (Figure 2F, 2P, 2U, Figure S7B).

The proteins Fis, H–NS, and the H–NS homologue StpA are encoded by the genes *fis*, *hns*, and *stpA*, and are conserved only in the proteobacteria. Hfq (*hfq*) is distributed from Gram-positive to Gram-negative species. Deletion of the *fis* gene resulted in a decrease in the populations of ”Intermediate” and “Thick” fibers and a dramatic increase in the population of ”Thin” fibers (Figure 2G, 2Q, 2U, Figure S7C). Deletion of the *hns* and *stpA* genes also led to an increase in the population of ”Thin” fibers (Figures 2H, 2I, 2R, 2S, 2U, Figure S7D, S8A). The populations of both ”Thin” and ”Thick” fibers increased in the Δ*hfq* mutant (Figure 2J, 2T, 2U, Figure S8B). Here, deletion of a gene encoding Fis, StpA or Hfq did not significantly affect the amount of H–NS. Similarly, the amount of Fis did not significantly change in Δ*fis*, Δ*stpA* and Δ*hfq* strains (Figure 3).

### Deletion of genes encoding major DNA-binding proteins does not result in exposure of naked DNA following RNase A treatment of lysed cells

**Figure 4:**
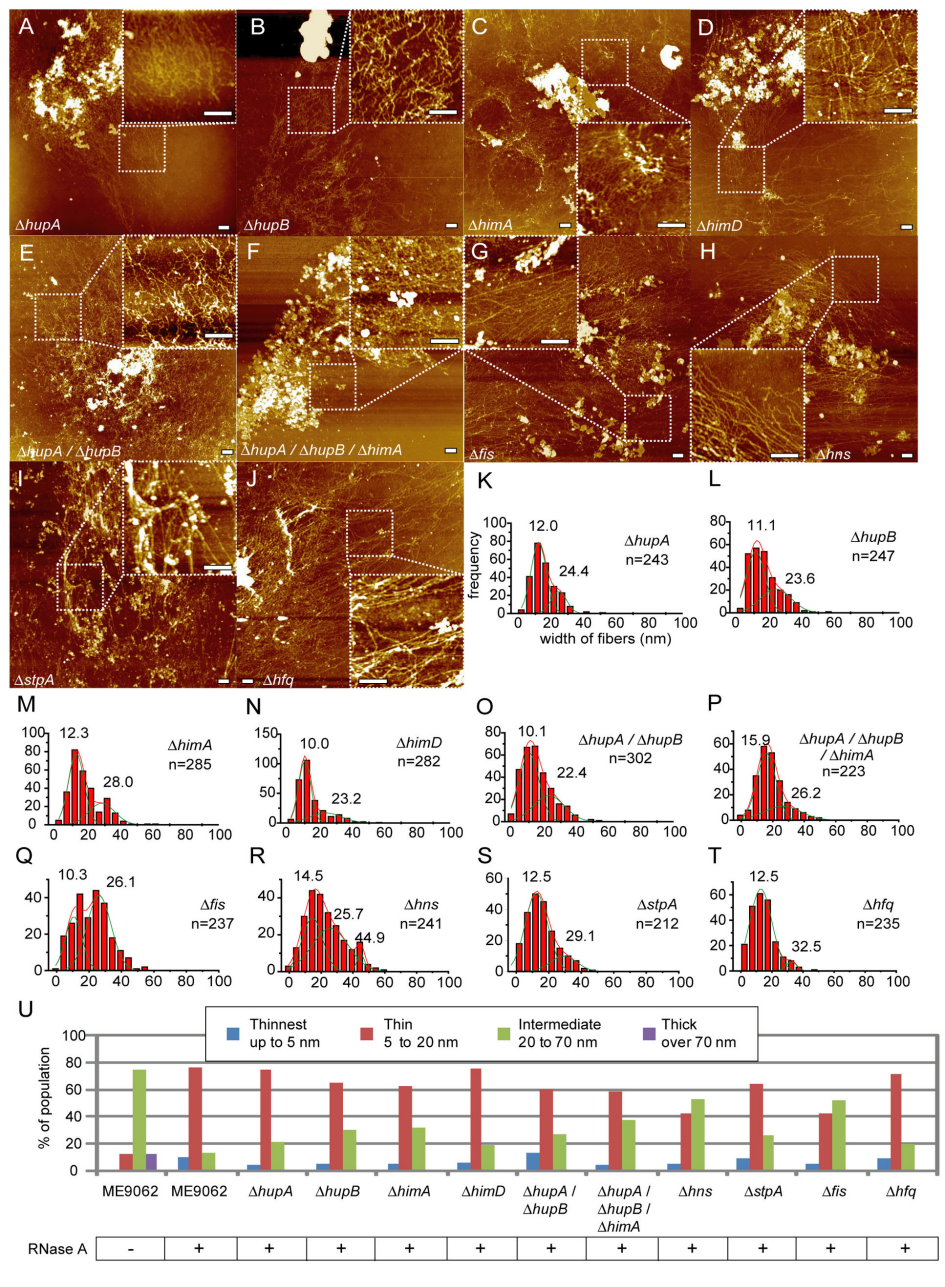
Treatment of deletion mutant nucleoids with RNase A. Lysed log phase *E*. *coli* cells were treated with RNase A. AFM images (A–J) and population distribution of fiber widths (K–T). Solid lines were obtained by Gaussian fitting, and the estimated peaks are as follows: (K) 12.0 ± 3.9 nm (mean ± SD) and 24.4 ± 4.0 nm (n = 243 total observations), (L) 11.1 ± 4.7 and 23.6 ± 7.9 nm (n = 247), (M) 12.3 ± 4.0 and 28.0 ± 6.7 nm (n = 285), (N) 10.0 ± 3.1 and 23.2 ± 8.3 nm (n = 282), (O) 10.1 ± 5.1 and 22.4 ± 8.1 nm (n = 302), (P) 15.9 ± 5.0 and 26.2 ± 9.7 nm (n = 223), (Q) 10.3 ± 3.6 and 26.1 ± 6.3 nm (n = 237), (R) 14.5 ± 1.9 nm, 25.7 ± 9.5 nm, and 44.9 ± 5.3 nm (n = 241), (S) 12.5 ± 5.8 and 29.1 ± 6.7 nm (n = 212), and (T) 12.5 ± 5.9 and 32.5 ± 2.8 nm (n = 235). (U) Categorization of fibers into 4 categories, “Thinnest”, “Thin”, “Intermediate” and “Thick”, as described in Figure 1. At least 5 nucleoids derived from 2 separate experiments were analyzed (Table S1). Scale bars in the AFM images represent 500 nm.

Since the deletion of genes encoding various major bacterial DNA-binding proteins did not result in the significant exposure of ”Thinnest” fibers in the above analyses, we hypothesized that none of a particular protein examined in this study are necessary for formation of the higher order structures from naked DNA. To test this hypothesis, we disrupted the nucleoid structure by treating lysed cells of each DNA-binding protein deletion mutant with RNase A, and observed whether naked DNA was exposed. Treatment of any of the single-deletion mutant strains with RNase A did not result in significant changes in the population of “Thinnest” fibers from the parental strain ME9062 (Figures 4A–D, 4G–N, 4Q–U, Figure S8C, S8D, S9A, S9B, S10A-D). Similarly, no significant change was observed following RNase A treatment of lysed double (*hupA*/*hupB*) or triple (*hupA*/*hupB/himA*) deletion mutant cells (Figures 4E, 4F, 4O, 4P, 4U, Figure S9C, S9D). These results confirmed that a particular DNA-binding protein we examined is dispensable for the formation of ”Thin” fibers from naked genomic DNA.

### Protease K and topoisomerase I convert the nucleoid into the naked DNA

**Figure 5:**
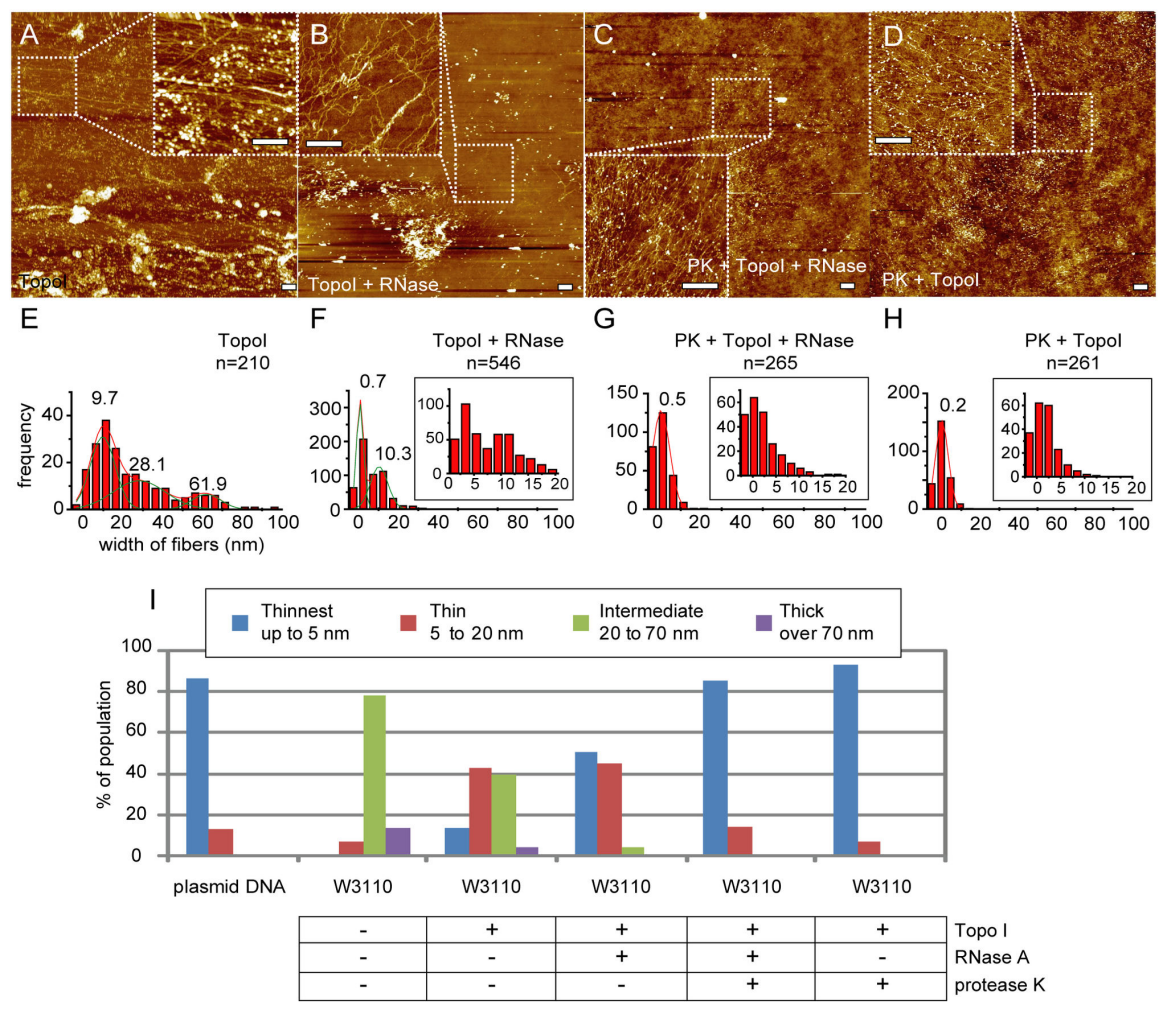
Treatment of nucleoids with topoisomerase I. Lysed log phase wild type *E*. *coli* W3110 cells were treated with topoisomerase I (Topo I) (A, E), Topo I and RNase A (B, F), Topo I and RNase A subsequent to protease K (C, G), and Topo I subsequent to protease K (D, H). AFM images (A–D) and population distribution of the widths of released fibers (E–H). Insets in (F), (G), and (H) show the population distributions with smaller bin size under 20 nm. Solid lines were obtained by Gaussian fitting, and the estimated peaks are as follows: (E) 9.7 ± 6.5 nm (mean ± SD), 28.1 ± 9.5 nm and 61.9 ± 5.3 nm (n = 210 total observations), (F) 0.7 ± 1.5 and 10.3 ± 3.7 nm (n = 546), (G) 0.5 ± 3.5 nm (n = 265), and (H) 0.2 ± 2.8 nm (n = 261). (I) Categorization of fibers into 4 categories, “Thinnest”, “Thin”, “Intermediate” and “Thick”, as described in Figure 1. At least 6 nucleoids derived from at least 2 separate experiments were analyzed (Table S1). Scale bars in the AFM images represent 500 nm.

Treatment with protease K resulted in exposed naked DNA, but ”Thin” and “Intermediate” fibers were still detected even after an extended period of protease K treatment (Figure 1D, 1L, 1Q, Figure S1D, S5C). This result suggests that factors other than proteins and RNAs are involved in the formation of "Thin" fibers from naked DNA, such as DNA supercoiling. Treatment of lysed cells with topoisomerase I (Topo I) and/or RNase A resulted in exposing a large population of "Thinnest" fibers (Figures 5A, 5B, 5E, 5F, 5I, Figure S5B, S5F, S11A, S11B). However, "Thin" fibers were still present even after treatment with an excess of both Topo I and RNase A, such that additional protease K treatment was required to completely disrupt the nucleoid structure into naked DNA (Figure 5C, 5G, 5I, Figure S11C). Here, RNase A treatment was not necessary to disrupt the nucleoid structure into naked DNA (Figure 5D, 5H, Figure S11D). These data indicate that the fibers in "Thin" category is composed of two different components, one of which is protease K-sensitive and another which is Topo I-sensitive. These components could not be distinguished by AFM analyses. It is also possible that components may be present that are sensitive to both protease K and Topo I (e.g., proteins that stabilize the DNA supercoiling might contribute to the formation of the fibers).

## Discussion

### Fibrous structures between 30 and 80 nm in diameter

We previously assumed that bacterial genomic DNA is folded step-wise to form the nucleoid as follows: DNA, 10-nm fiber, 30-nm fiber, 80-nm fibers/beads, condensed nucleoid [13–17]. In this study, we applied the "grid-analysis" technique to objectively evaluate the population of fiber widths and demonstrated the presence of intermediate populations (e.g., 50-nm diameter fibers, Figure 1) in both wild type *E. coli* and the parental strain used to prepare deletion mutants. Here, the histogram peaks indicative of populations of fibers between 30-nm and 80-nm diameter were not clearly separated. This difference was likely due to the method used to select the target to be measured. We previously selected fibrous structures of homogeneous thickness, and excluded irregular portions such as blobs. However, many fibers in “Intermediate” category were not smooth and of homogenous width, but instead contained various regions of irregular shape. In this study, we measured all portions of the fibrous structures, which accounts in part for the accumulation of intermediate populations. The heterogeneous configuration of proteins along the genomic DNA may cause such a heterogeneous appearance in a single fiber structure. Although the 50-nm population of fibers was suggested from our peak fit analyses, it is possible that the thickness of the structure classified in "Intermediate" fibers is heterogeneous. Together with the observation that strains containing various deletions (Δ*hupA*, Δ*hupA*/Δ*hupB*/Δ*himA*, and Δ*hfq*) exhibited a peak indicative of a population of fibers closer to 60 nm than to 50 nm (Figure 2), our results suggest that the fibrous structures in "Intermediate" category consist of fibers of variable thickness rather than a few types of homogeneous and smooth fibers.

It is possible that the existence of fibrous structures reflects transcription and translation taking place in the area surrounding the nucleoid [19]. This possibility is supported by the fact that treating wild type cells in culture with rifampicin or chloramphenicol resulted in a breakdown of the larger fibrous structures (Figure 1) [16]. Thus, it is likely that fibers categorized in “Intermediate” and “Thick” consist of a heterogeneous and dynamic complex of RNA, DNA, RNA polymerases, and ribosomes in addition to DNA-binding proteins.

### Involvement of major DNA-binding proteins in the nucleoid architecture

A single log phase *E. coli* cell contains about 50,000 HU molecules, including both HUα and HUβ capable of forming homo- and heterodimers [20,21]. Interestingly, deletion of the *hupA* gene resulted in an increase in the population of “Thin” fibers, while deletion of *hupB* led to increases in the population of “Thick” fibers (Figure 2). The opposing effect resulting from deletion of the HU genes may be related to distinct transcriptional activity in the Δ*hupA* and Δ*hupB* mutants [22]. In contrast to the case of the HU-deletion mutants, the population of “Thin” fibers was increased in both the Δ*himA* and Δ*himD* mutants (Figure 2).

The Δ*hupA*/Δ*hupB* double knockout mutant exhibited a larger population of “Thick” fibers than did the Δ*hupA* and Δ*hupB* mutants or the parental strain, but the reason for this difference is unknown. Deletion of the *himA* gene in the Δ*hupA*/Δ*hupB* background (Δ*hupA*/Δ*hupB*/Δ*himA* triple mutant) resulted in an increase in the population of “Thin” fibers, as was the case with the Δ*himA* single mutant. The level of HU protein did not decrease in the Δ*himA* or Δ*himD* mutants (Figure 3), suggesting that the effect of IHF on nucleoid structure is, at least in part, independent of HU. Integration host factor IHF is a HU homologue, but unlike HU, IHF is known to prefer a specific DNA sequence [7]. In general, *E. coli* cells express about 5 times more HU than IHF [20], but IHF may be localized at specific loci in the genome, where it plays an important role in sustaining the nucleoid structure.

The frequency of “Thin” fibers in the mutants lacking one of several “abundant” DNA-binding proteins correlates well with the quantity of these proteins in a normal cell (Fis, about 60,000 molecules; StpA, about 25,000 molecules, and H–NS, about 20,000 molecules [20]). The lack of a particular one is not likely to affect the amount of Fis or H–NS (Figure 3). It should be noted that Fis, H–NS, and StpA preferably bind to A/T-rich regions as global transcription regulators [8–12]. The A/T preference is not shared with HU and IHF. It is possible that Fis, H–NS, and StpA contribute to the structure of the nucleoid at distinct loci from those bound by HU and IHF. Whether these proteins function independently or in cooperation with HU/IHF in sustaining the nucleoid structure remains unknown.

### The 10-nm fiber as the basic component of higher-order structures

**Figure 6:**
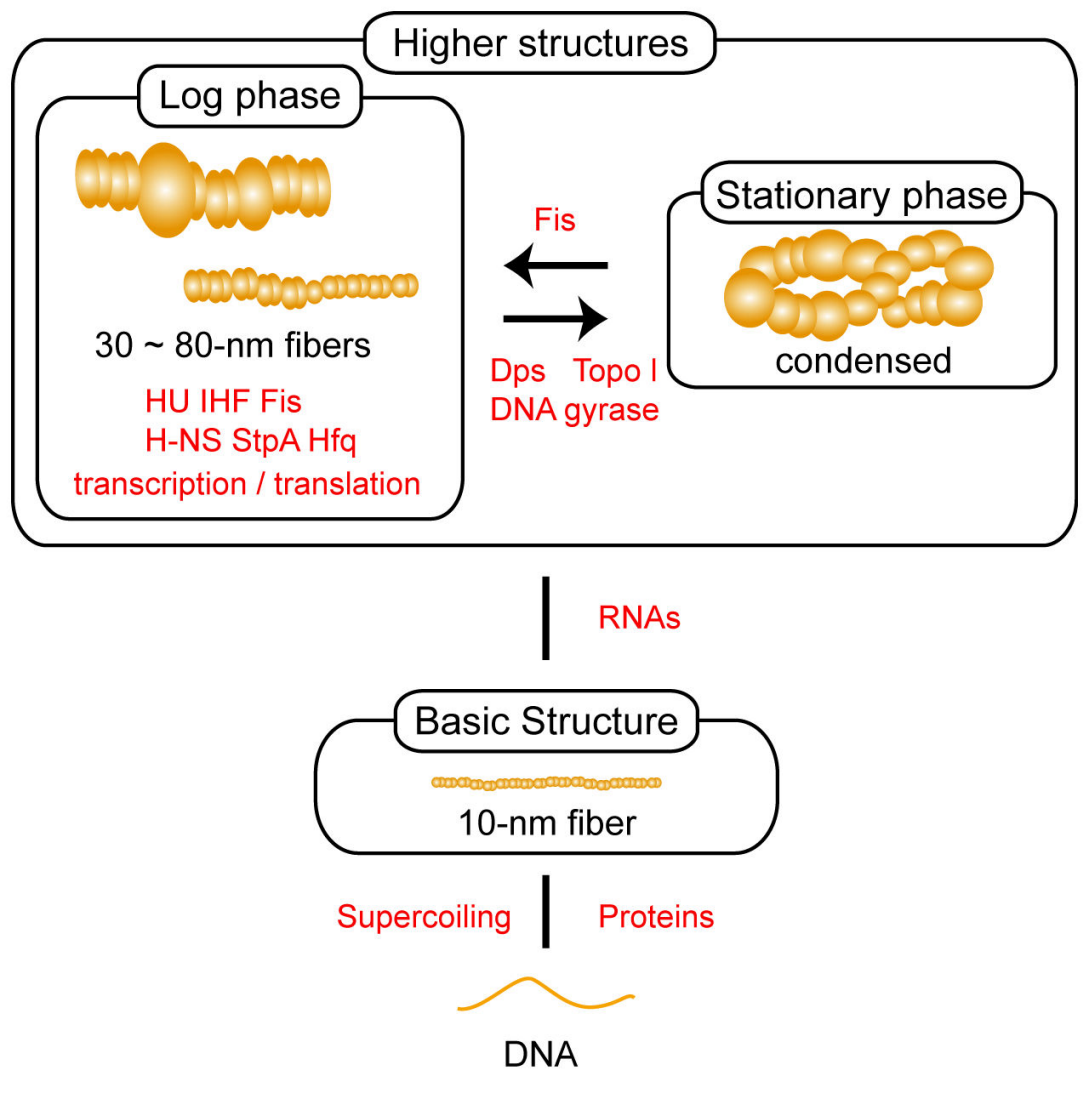
A model of nucleoid organization in *E. coli*. Proteins and DNA supercoiling are important in formation of 10-nm fibers. The proteins involved may include major nucleoid proteins (HU, IHF, H–NS, StpA, Fis, and Hfq), although the lack of any one of these proteins can be compensated for by other nucleoid proteins. The major nucleoid proteins sustain fibers with a diameter of 30 nm to 80 nm. Fibers with a diameter between 30 nm and 80 nm are heterogeneous, or rugged, in their thickness. Proteins involved in transcription and translation also participate in sustaining the structures. The transition between the fiber structures in log phase cells and the condensed structure observed in stationary phase cells is regulated by Fis, Dps, Topo I, and DNA gyrase [24]. The expression of Dps with Topo I and DNA gyrase as cells progress toward stationary phase induces nucleoid condensation, while Fis inhibits nucleoid condensation by stabilizing the DNA topology during log phase growth. RNA is required to sustain the condensed structure [16], as well as 30~80-nm fibers.

The results of the present AFM investigation of the structure of the nucleoid prompted us to revise the structural model of the nucleoid (Figure 6). Here, naked DNA is initially folded into 10-nm fibers by certain proteins and/or DNA supercoiling, but formation of the 10-nm fibers does not require HU, IHF, Fis, H–NS, StpA, or Hfq, although it is undeniable that the lack of any of these major nucleoid protein is compensated for by other nucleoid proteins. Here, it might be possible that these proteins participate the modulation of the 10-nm fiber structure, because the lack of a particular protein changed the peaks in “Thin” fiber population in the range of 8.1-16.3 nm (Figure 2 and Figure 3). The 10-nm fiber consists of protease K-sensitive and topoisomerase I-sensitive regions. Under our experimental conditions, we could not identify any intermediate population of fibers between naked DNA and the 10-nm fiber. The 10-nm fiber is thus likely the basic unit from which the higher-order structures are formed in a process that requires various DNA-binding proteins in addition to molecules involved in transcription and translation. Our conclusion that the 10-nm fiber is the basic structure is also supported by the fact that the condensed nucleoid in stationary phase cells is disrupted into 10-nm fibers upon RNase A treatment [16].

## Materials and Methods

### Bacterial strains and culture conditions

The *E. coli* K-12 strains used in the study are summarized in Table 1. Mutant strains lacking a single gene such as *hupA*, *hupB*, *himA*, *himD*, *fis*, *hns*, *stpA*, or *hfq* were derived from *E. coli* K-12 strain ME9062 [23]. The double and triple deletion mutants Δ*hupA/*Δ*hupB* and Δ*hupA/*Δ*hupB/*Δ*himA* were derived from *E. coli* K-12 strain YK1100. Glycerol stocks of *E. coli* strains were inoculated into LB medium and cultured at 37° C with constant shaking (180 rpm, Bioshaker BR-15; TAITEC, Tokyo, Japan) for 18 h. A 50 µL aliquot of the over night culture was then inoculated into 5 mL of fresh LB medium and cultured at 37° C with constant shaking (180 rpm, Bioshaker BR-15) until the OD_600_ reached 0.5. If necessary, rifampicin or chloramphenicol was added to the log phase culture to inhibit transcription or translation, respectively (final concentration 100 μg/mL), and the cultures were incubated for an additional 60 min at 37° C. The cell density was determined by measuring the absorbance at 600 nm using a UV-160A spectrophotometer (Shimadzu, Tokyo, Japan).

### Cell lysis

Bacterial cells were harvested from 100-μL cultures by centrifugation (13,000 x *g*, 1 min, 4° C) and washed once with 1 mL of phosphate buffered saline (PBS, pH 7.2). The cells were resuspended in 250 μL of PBS and a 50-μL aliquot was placed on a round cover glass (15 mm in diameter). Excess liquid was removed by nitrogen gas evaporation. The cover glass was then immersed in 2 mL of buffer (10 mM Tris-HCl, pH 8.2, 1 mM NaN_3_, and 0.1 M NaCl) for 5 min, after which a solution containing 25 μg/mL of lysozyme was added. After incubation for 2 min at 25° C, Brij 58 (polyoxyethylene hexadecyl ether) and sodium deoxycholate were added to final concentrations of 0.25 mg/mL and 0.1 mg/mL, respectively. After 10 min, the cover glass was dried under nitrogen gas and the surface was gently washed with distilled water and dried again. The cells were then treated with distilled water, 5 μg/mL DNase-free RNase A (Roche, UK), 1 mg/mL RNA-grade protease K (Invitrogen, USA), and/or 4 U of topoisomerase I (Promega, USA) for 60 min or 120 min at 37° C. Finally, the surface of the cover glass was washed and dried.

### Microscopy

The nucleoid of lysed *E. coli* cells were examined in air at room temperature by atomic force microscopy using Seiko SP3800N or SPI400 (Seiko Instruments, Tokyo) or Bruker Nanoscope VIII (Bruker ASX, Germany) AFM instruments. The systems were operated in tapping mode with a 100-μm scanner. Probes made of a single silicon crystal with a cantilever length of 129 µm and spring constant of 33-62 N/m (OMCL-AC160TS-W2, Olympus, Japan) were used for imaging. Data were collected in the height mode. Images were captured in at least 512 × 512 pixel format and the captured images were flattened and plane-fitted before analysis. Software provided with the imaging module was used to analyze the images.

### Data analysis

To objectively evaluate the population distribution of the width of fibrous structures, it is critical to account for person-to-person variations in picking up the fibrous structures to be measured. This was accomplished by measuring the fibrous structures on 8 x 8 lines drawn at regular intervals with 250 nm on a 2 μm x 2 μm scale image ("grid-analysis", Figure S2).

The apparent horizontal dimensions of samples measured using AFM are generally much larger than the real dimensions due to the effect of edge curvature and the point angle of the cantilever. We estimated the real dimension of the fibers using the “circular cone model” introduced by Ohniwa et al. in 2007 (Figure S3) [16]. Briefly, when a sample with a real dimension larger than 10 nm is imaged using an OMCL-AC160TS-W2 cantilever, the real dimension of the sample (S) can be determined from the equation S (nm) = 0.75x W (nm) - 16.14, where W represents the apparent width of the sample. These parameter values (0.75 and 16.14) are obtained from measurements of 10-nm, 30-nm, and 80-nm gold particles [16]. When the real sample size is smaller than 10 nm, this estimation gives a slightly smaller dimension than the real one due to the different mode of contact between the cantilever and the sample (i.e., samples smaller than 10 nm contact the curved surface of the tip while samples lager than 10 nm contact a side face of the cantilever (Figure S3). The population distribution of fiber widths was evaluated by constructing histograms with subsequent multiple Gaussian fit analysis using Origin 5.0 software (Light Stone, Japan).

## Supporting Information

Figure S1 **Enlarged AFM images shown in Figures 1A, 1B, 1C and 1D.**
Lysed log phase *E. coli* W3110 (A) and ME9062 (B) cells. Lysed log phase W3110 cells were treated with RNase A (C) or protease K (D).
(TIF)

Figure S2 **Scheme illustrating grid analysis of AFM images.**
(A, B) In the grid analysis process, 8 x 8 lines at regular intervals with 250 nm are drawn on each image taken at a scale of 2 μm x 2 μm with at least 512 x 512 pixel quality. The apparent width of fibers on the drawn lines are then measured (positions represented by cross marks in the left pane A). This procedure may eliminate the risk of bias in the selection of samples to be measured (cross- marks in panel B). We measured the fiber widths of three zoomed-up images (D, E, F) taken from the image C by applying both grid analysis (G, H, I, J, K, L) and manual selection of fibers (M, N, O). Image D corresponds to graphs G, J and M, Image E to graphs H, K and N, and Image F to graphs I, L and O, respectively. Histograms G, H and I were obtained when the grid was fixed at the original position as represented in (A). Histograms J, K and L were obtained after moving the grid for a half size of square from the original position. (P) Categorization of fibers into 4 categories, “Thinnest”, “Thin”, “Intermediate” and “Thick”, as described in Figure 1. Black lines in images D, E and F indicate sections for the manual selection. The manual selection of fibers exaggerated the population of “Thin” fibers (arrow heads in M, N, and P), which were proven as minor population by the grid-analysis (G, H, I and J).
(TIF)

Figure S3 **Circular cone model used to eliminate the tip effect [16].**
(A) Illustration of the relationship between the cantilever tip and sample when the sample size is smaller than the tip (*Y* > *H*, where *Y* represents the distance between the surface and the end of the curvature, and *H* represents the distance between the surface and the contact point between the sample and the tip). (B) Illustration of the relationship between the cantilever tip and sample when the sample size is larger than the tip (*Y* < *H*). (C) Illustration of the relationship between the cantilever tip and sample when the edge of the tip is an ideal incircle. Based upon electron microscopic images of the tip (OMCL-AC160TS-W2, Olympus) (Catalog: http://probe.olympus-global.com/en/), the geometry of the cantilever and a globular sample can be drawn as in (A) or (B). The relationship between the apparent width of the sample in the image (*W*), the radius of curvature of the tip (*Rc*), the real radius of the sample (*Rm*), and the point angle of the tip (2 x θ) can be given as follows:.
*W*=4×(*Rc*×*Rm*)^1/2^, *Y* > *H* (I) [25]
*W*=2×{*N*+*Rm*×(1+*sinθ*)/*cosθ*} *Y* < *H* (II)
H=Rm×(sinθ+1) (III)
The *N* term in equation (II) represents the distance that depends on the shape of the tip edge. Equation (III) provides the value used to determine whether equation (I) or (II) should be applied to estimate *W* and *Rm*. In the case of naked DNA, since the *Rm* of DNA is 1 nm and the average θ of the tip is 12.5^°^ (from the catalog) PLoSONErevise3rd_proof3.docx, *H* is 1.2 nm. If the edge of the tip is an ideal incircle of the circular cone (C), Y is *Rc* x (1 – sin θ). In this case, Y is 5.9 nm for *Rc* = 7.5 nm and θ= 12.5^°^ (from the catalog). Therefore, small samples such as DNA should be evaluated using equation (I). In contrast, the *H* of a nucleosome in eukaryotic chromosomes is 6.7 using an *Rm* = 5.5 nm as determined from the X-ray crystal structure of the nucleosome [26], and θ= 12.5^°^; therefore, samples larger than the nucleosome should be evaluated using equation (II). Since equation (II) is a linear function and the real width of the sample (*S*) is 2 x *Rm*, equation (II) becomes:
S=2×Rm=A×B (IV)
where *A* and *B* are constants determined by the tip characteristics (*N* and θ). Values for *A* and *B* can be obtained using a standard sample such as gold nano-particles.
(TIF)

Figure S4 **Enlarged AFM images shown in Figures 1E, 1F, 1G and 1H.**
Lysed log phase W3110 cells were treated with RNase A subsequent to protease K (A). Log phase W3110 cells were treated in culture with 100 μg/mL of rifampicin (B) or 100 μg/mL of chloramphenicol (C) for 60 min, and were lysed. (D) AFM image of naked plasmid DNA (pRSFDuet-1, 3829 bp, Novagen).
(TIF)

Figure S5 **Extended duration treatment with distilled water, RNase A, protease K, and topoisomerase I.**
Lysed log phase *E. coli* W3110 cells were treated with distilled water, RNase A, protease K, or topoisomerase I for 120 min. AFM images (A–D) and the population distribution of fiber widths (E–H) are shown. Solid lines were obtained by Gaussian fitting, and the estimated peaks are as follows: (E) 12.0 ± 3.9 nm (mean ± SD) and 24.4 ± 4.0 nm (n = 243 total observations), (F) 7.5 ± 3.6, 24.5 ± 8.9, and 56.5 ± 7.8 nm (n = 218), (G) 3.9 ± 3.4, 14.5 ± 1.3, and 27.7 ± 5.4 nm (n = 175), and (H) 9.7 ± 3.6 and 25.5 ± 5.9 nm (n = 185). At least 5 nucleoids derived from 2 separate experiments were analyzed. Scale bars in the AFM images represent 500 nm.
(TIF)

Figure S6 **Enlarged AFM images shown in Figures 2A, 2B, 2C and 2D.**
Lysed log phase *E. coli* Δ*hupA* (A), Δ*hupB* (B), Δ*himA* (C) and Δ*himD* (D) cells.
(TIF)

Figure S7 **Enlarged AFM images shown in Figures 2E, 2F, 2G and 2H.**
Lysed log phase *E. coli* Δ*hupA*/Δ*hupB* (A), Δ*hupB/*Δ*hupB/*Δ*himA* (B), Δ*fis* (C) and Δ*hns* (D) cells.
(TIF)

Figure S8 **Enlarged AFM images shown in Figures 2I, 2J, 3A and 3B.**
Lysed log phase *E. coli* Δ*stpA* (A), Δ*hfq* (B), Δ*hupA* treated with RNase A (C) and Δ*hupB* treated with RNase A (D) cells.
(TIF)

Figure S9 **Enlarged AFM images shown in Figures 3C, 3D, 3E and 3F.**
RNase A treated lysed log phase *E. coli* Δ*himA* (A), Δ*him* (B), Δ*hupA*/Δ*hupB* (C) and Δ*hupB/*Δ*hupB/*Δ*himA* (D) cells.
(TIF)

Figure S10 **Enlarged AFM images shown in Figures 3G, 3H, 3I and 3J.**
RNase A treated lysed log phase *E. coli* Δfis (A), Δ*hns* (B), Δ*stpA* (C) and Δ*hfq* (D) cells.
(TIF)

Figure S11 **Enlarged AFM images shown in Figures 5A, 5B, 5C and 5D.**
Lysed *E. coli* W3110 cells treated with topoisomerase I (A), topoisomerase I and RNase A (B), topoisomerase I, RNase A and protease K (C), and topoisomerase I and protease K (D).
(TIF)

Table S1 **Number of experiments and observed cells.**
(XLSX)
