# Supplementary figures and images for: Atomic Force Microscopy Analysis of the Role of Major DNA-Binding Proteins in Organization of the Nucleoid in Escherichia coli
Source: PLoS One. 2013 Aug 12;8(8):e72954. doi: 10.1371/journal.pone.0072954 (PMC3741201; doi:10.1371/journal.pone.0072954)

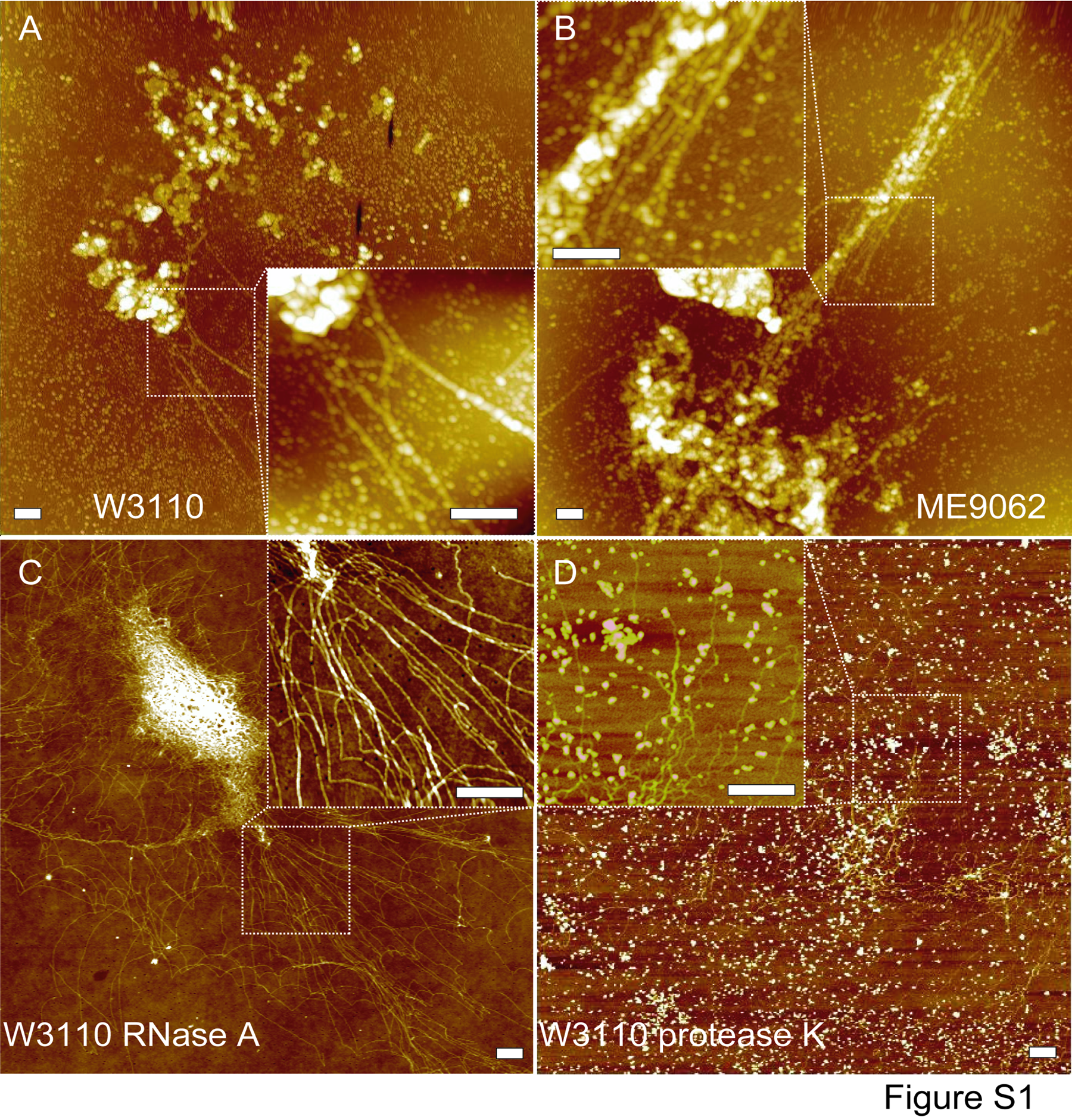

Supplement: Figure S1 — Enlarged AFM images shown in Figures 1A, 1B, 1C and 1D. Lysed log phase E. coli W3110 (A) and ME9062 (B) cells. Lysed log phase W3110 cells were treated with RNase A (C) or protease K (D). (TIF) [file pone.0072954.s001.tif]

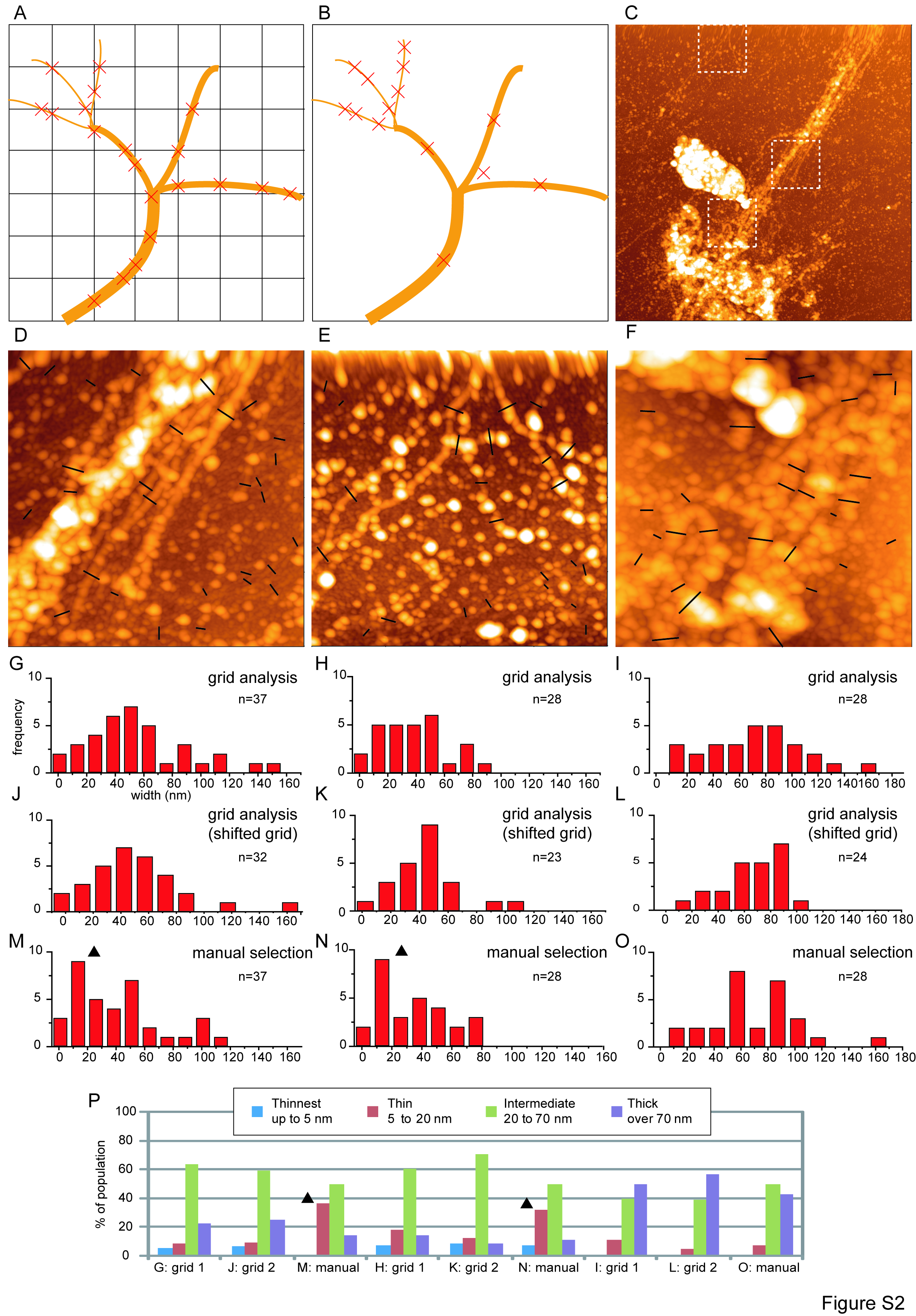

Supplement: Figure S2 — Scheme illustrating grid analysis of AFM images. (A, B) In the grid analysis process, 8 x 8 lines at regular intervals with 250 nm are drawn on each image taken at a scale of 2 μm x 2 μm with at least 512 x 512 pixel quality. The apparent width of fibers on the drawn lines are then measured (positions represented by cross marks in the left pane A). This procedure may eliminate the risk of bias in the selection of samples to be measured (cross- marks in panel B). We measured the fiber widths of three zoomed-up images (D, E, F) taken from the image C by applying both grid analysis (G, H, I, J, K, L) and manual selection of fibers (M, N, O). Image D corresponds to graphs G, J and M, Image E to graphs H, K and N, and Image F to graphs I, L and O, respectively. Histograms G, H and I were obtained when the grid was fixed at the original position as represented in (A). Histograms J, K and L were obtained after moving the grid for a half size of square from the original position. (P) Categorization of fibers into 4 categories, “Thinnest”, “Thin”, “Intermediate” and “Thick”, as described in Figure 1. Black lines in images D, E and F indicate sections for the manual selection. The manual selection of fibers exaggerated the population of “Thin” fibers (arrow heads in M, N, and P), which were proven as minor population by the grid-analysis (G, H, I and J). (TIF) [file pone.0072954.s002.tif]

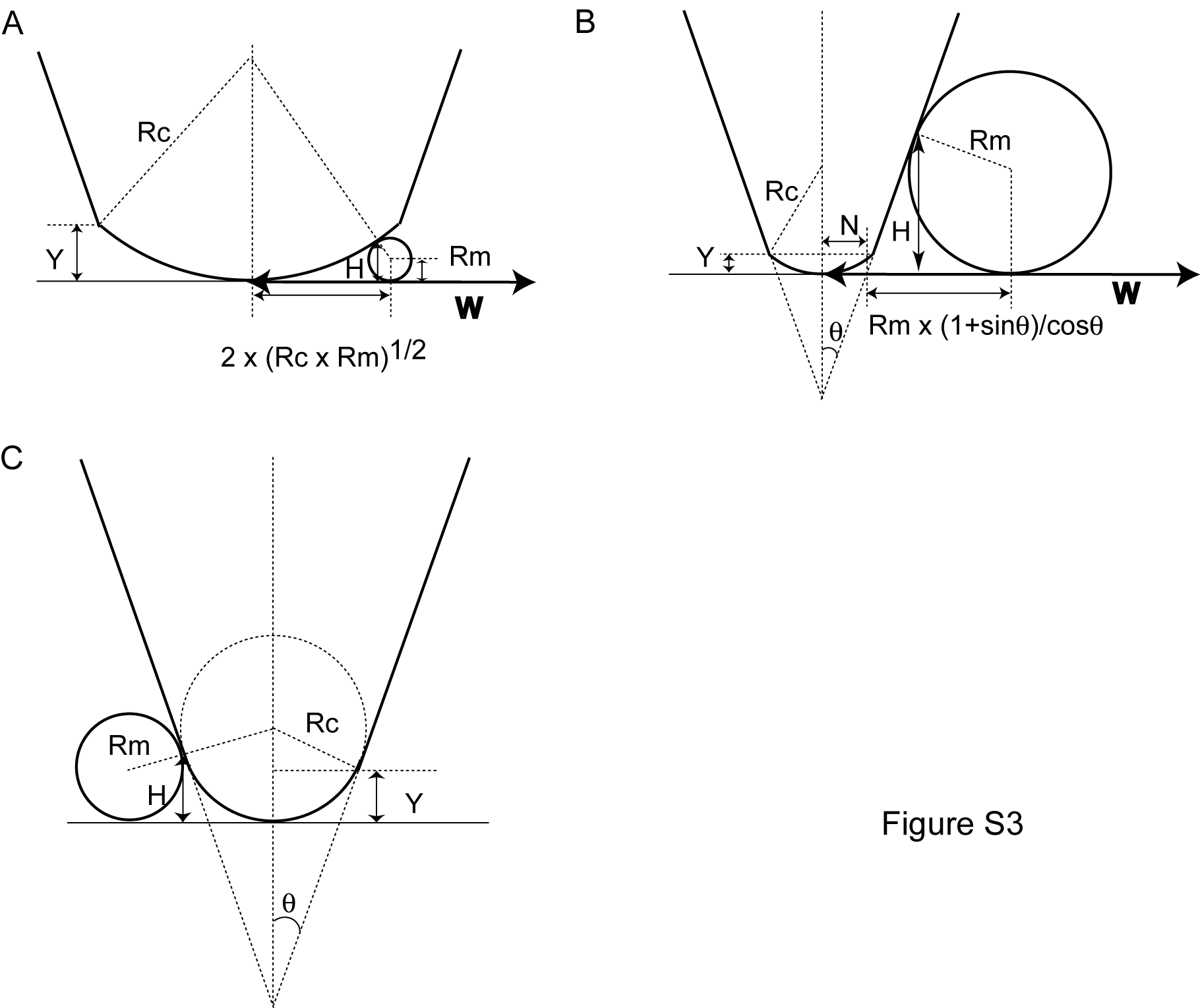

Supplement: Figure S3 — Circular cone model used to eliminate the tip effect [16]. (A) Illustration of the relationship between the cantilever tip and sample when the sample size is smaller than the tip (Y > H, where Y represents the distance between the surface and the end of the curvature, and H represents the distance between the surface and the contact point between the sample and the tip). (B) Illustration of the relationship between the cantilever tip and sample when the sample size is larger than the tip (Y < H). (C) Illustration of the relationship between the cantilever tip and sample when the edge of the tip is an ideal incircle. Based upon electron microscopic images of the tip (OMCL-AC160TS-W2, Olympus) (Catalog: http://probe.olympus-global.com/en/), the geometry of the cantilever and a globular sample can be drawn as in (A) or (B). The relationship between the apparent width of the sample in the image (W), the radius of curvature of the tip (Rc), the real radius of the sample (Rm), and the point angle of the tip (2 x θ) can be given as follows:. W=4×(Rc×Rm)1/2, Y > H (I) [25] W=2×{N+Rm×(1+sinθ)/cosθ} Y < H (II) H=Rm×(sinθ+1) (III) The N term in equation (II) represents the distance that depends on the shape of the tip edge. Equation (III) provides the value used to determine whether equation (I) or (II) should be applied to estimate W and Rm. In the case of naked DNA, since the Rm of DNA is 1 nm and the average θ of the tip is 12.5° (from the catalog) PLoSONErevise3rd_proof3.docx, H is 1.2 nm. If the edge of the tip is an ideal incircle of the circular cone (C), Y is Rc x (1 – sin θ). In this case, Y is 5.9 nm for Rc = 7.5 nm and θ= 12.5° (from the catalog). Therefore, small samples such as DNA should be evaluated using equation (I). In contrast, the H of a nucleosome in eukaryotic chromosomes is 6.7 using an Rm = 5.5 nm as determined from the X-ray crystal structure of the nucleosome [26], and θ= 12.5°; therefore, samples larger than the nucleosome should be evaluated using e [file pone.0072954.s003.tif]

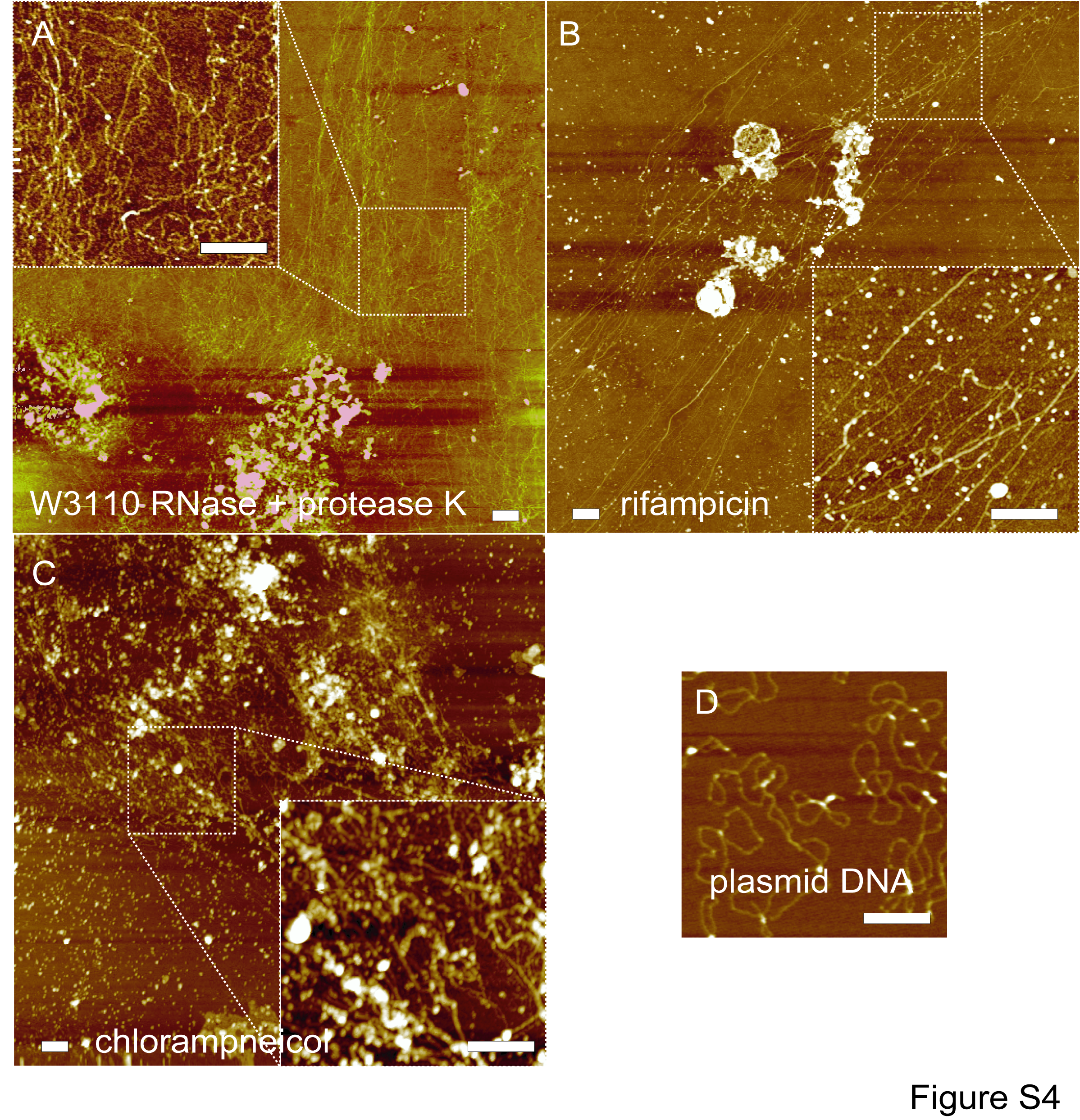

Supplement: Figure S4 — Enlarged AFM images shown in Figures 1E, 1F, 1G and 1H. Lysed log phase W3110 cells were treated with RNase A subsequent to protease K (A). Log phase W3110 cells were treated in culture with 100 μg/mL of rifampicin (B) or 100 μg/mL of chloramphenicol (C) for 60 min, and were lysed. (D) AFM image of naked plasmid DNA (pRSFDuet-1, 3829 bp, Novagen). (TIF) [file pone.0072954.s004.tif]

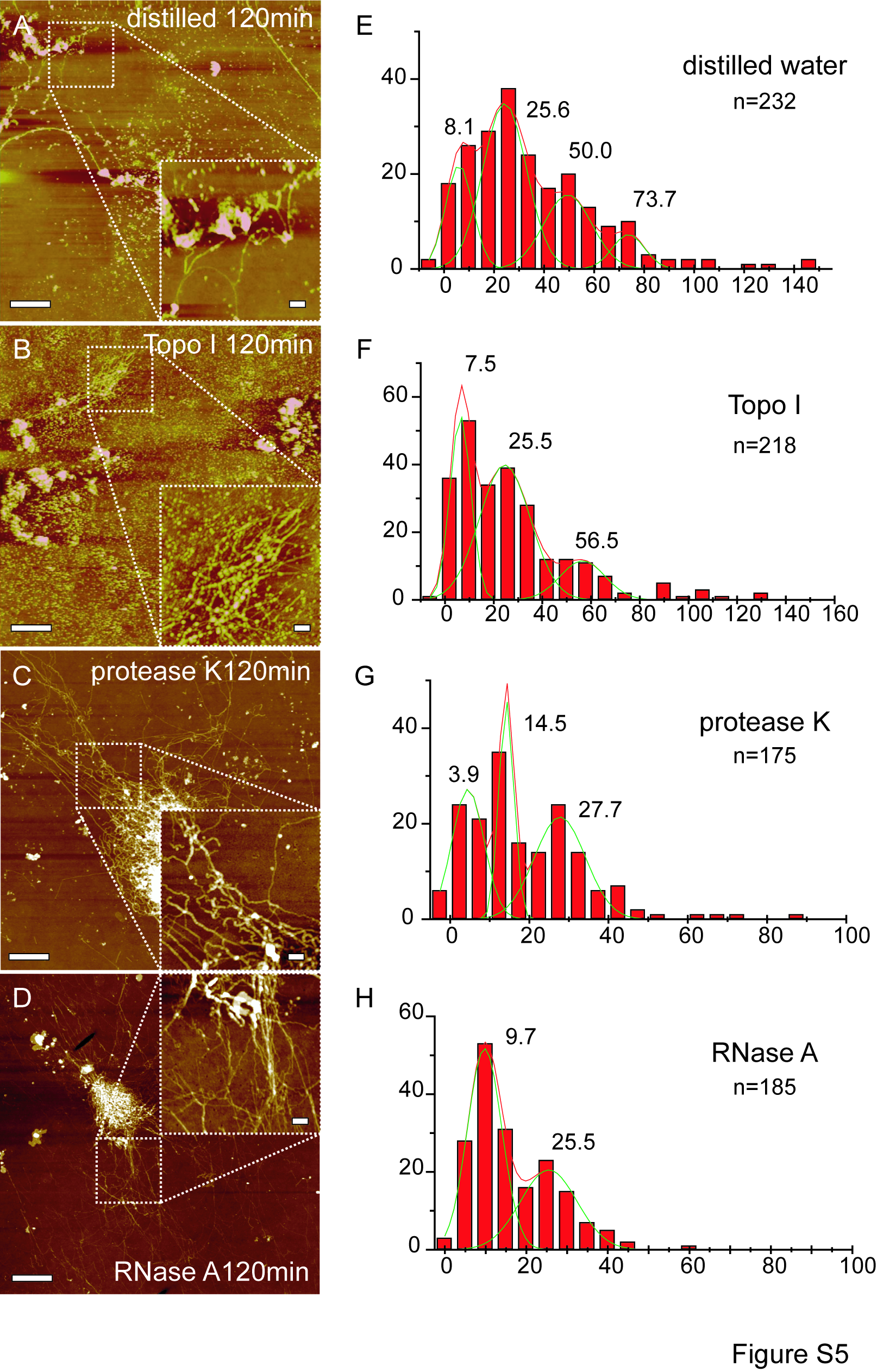

Supplement: Figure S5 — Extended duration treatment with distilled water, RNase A, protease K, and topoisomerase I. Lysed log phase E. coli W3110 cells were treated with distilled water, RNase A, protease K, or topoisomerase I for 120 min. AFM images (A–D) and the population distribution of fiber widths (E–H) are shown. Solid lines were obtained by Gaussian fitting, and the estimated peaks are as follows: (E) 12.0 ± 3.9 nm (mean ± SD) and 24.4 ± 4.0 nm (n = 243 total observations), (F) 7.5 ± 3.6, 24.5 ± 8.9, and 56.5 ± 7.8 nm (n = 218), (G) 3.9 ± 3.4, 14.5 ± 1.3, and 27.7 ± 5.4 nm (n = 175), and (H) 9.7 ± 3.6 and 25.5 ± 5.9 nm (n = 185). At least 5 nucleoids derived from 2 separate experiments were analyzed. Scale bars in the AFM images represent 500 nm. (TIF) [file pone.0072954.s005.tif]

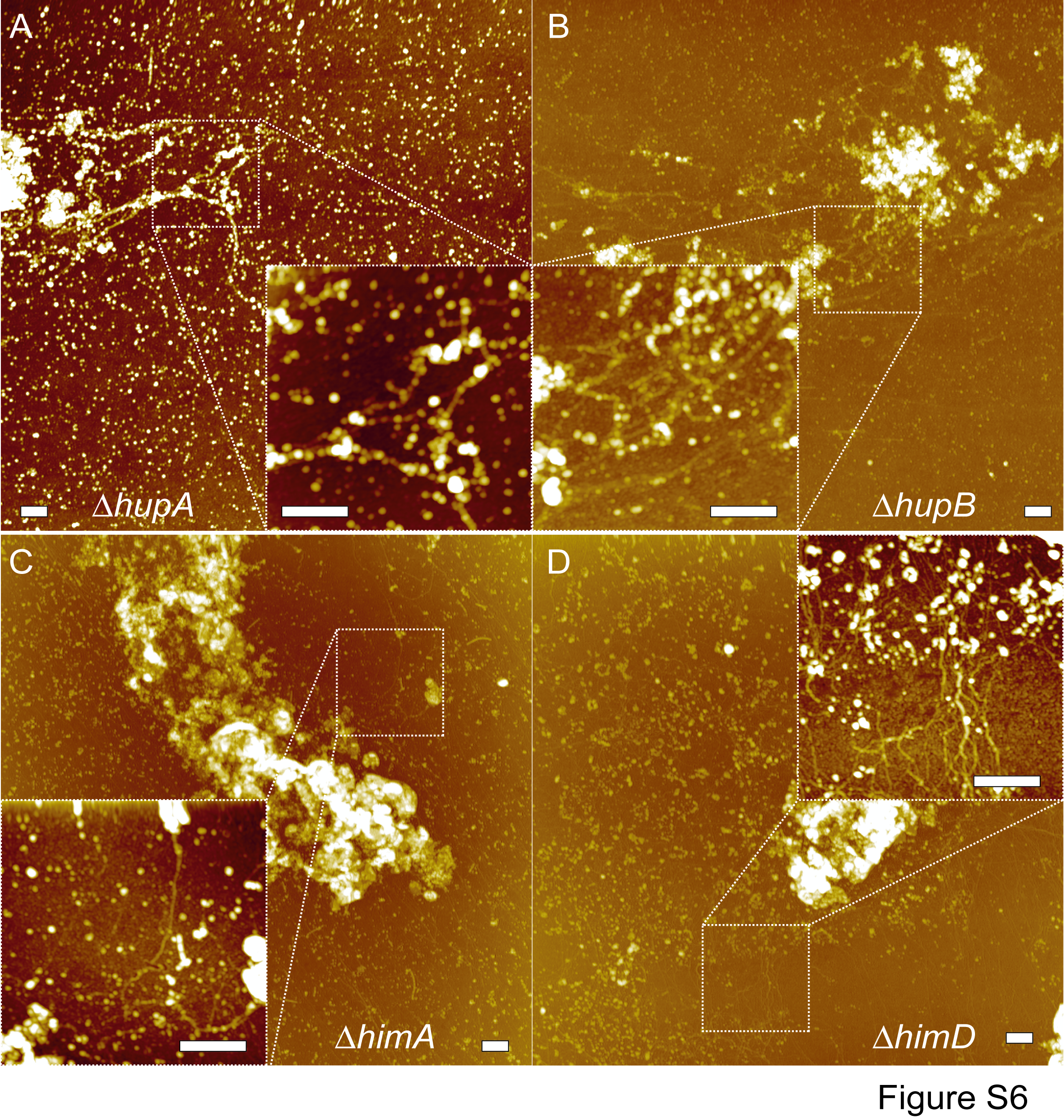

Supplement: Figure S6 — Enlarged AFM images shown in Figures 2A, 2B, 2C and 2D. Lysed log phase E. coli ΔhupA (A), ΔhupB (B), ΔhimA (C) and ΔhimD (D) cells. (TIF) [file pone.0072954.s006.tif]

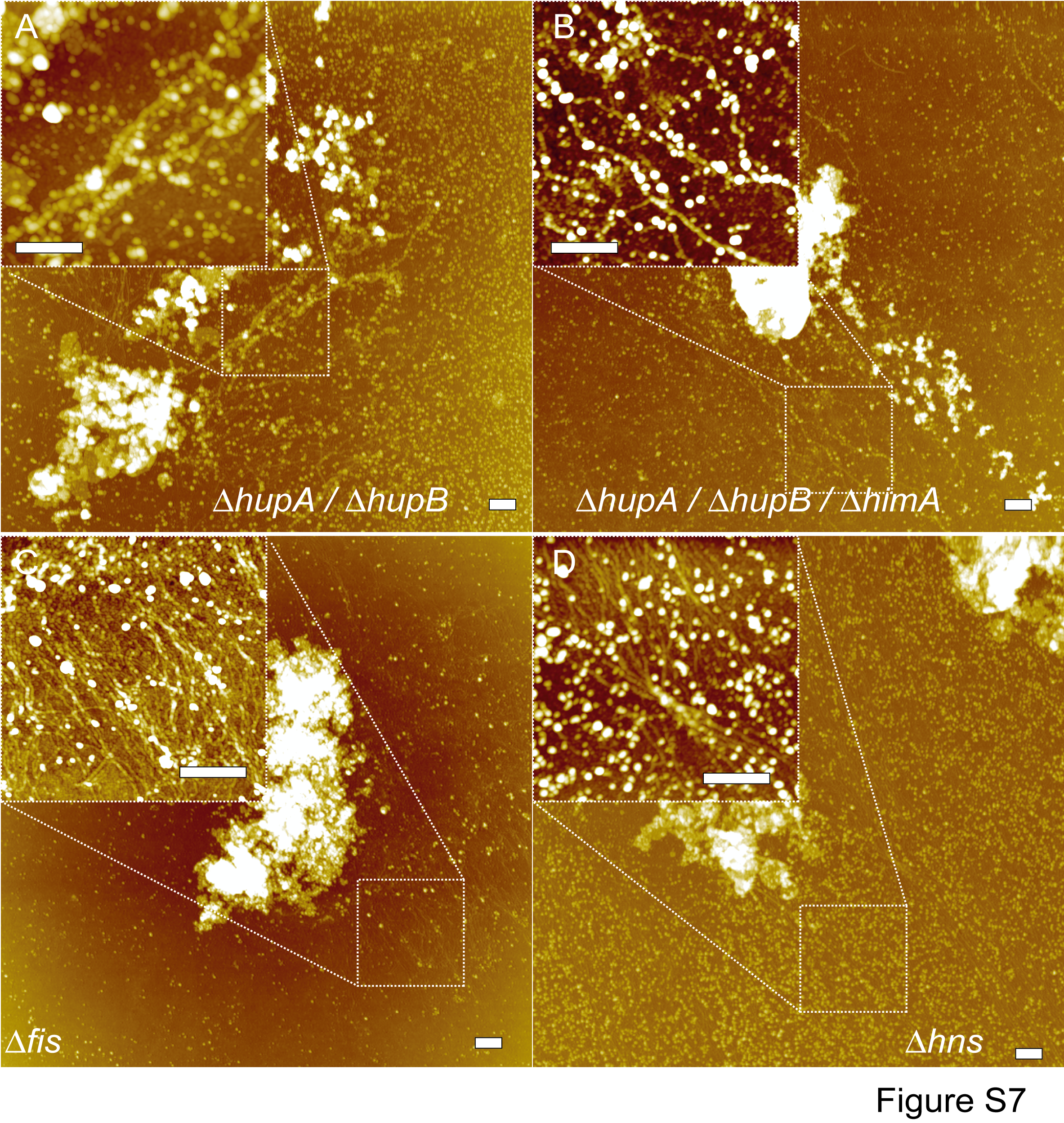

Supplement: Figure S7 — Enlarged AFM images shown in Figures 2E, 2F, 2G and 2H. Lysed log phase E. coli ΔhupA/ΔhupB (A), ΔhupB/ΔhupB/ΔhimA (B), Δfis (C) and Δhns (D) cells. (TIF) [file pone.0072954.s007.tif]

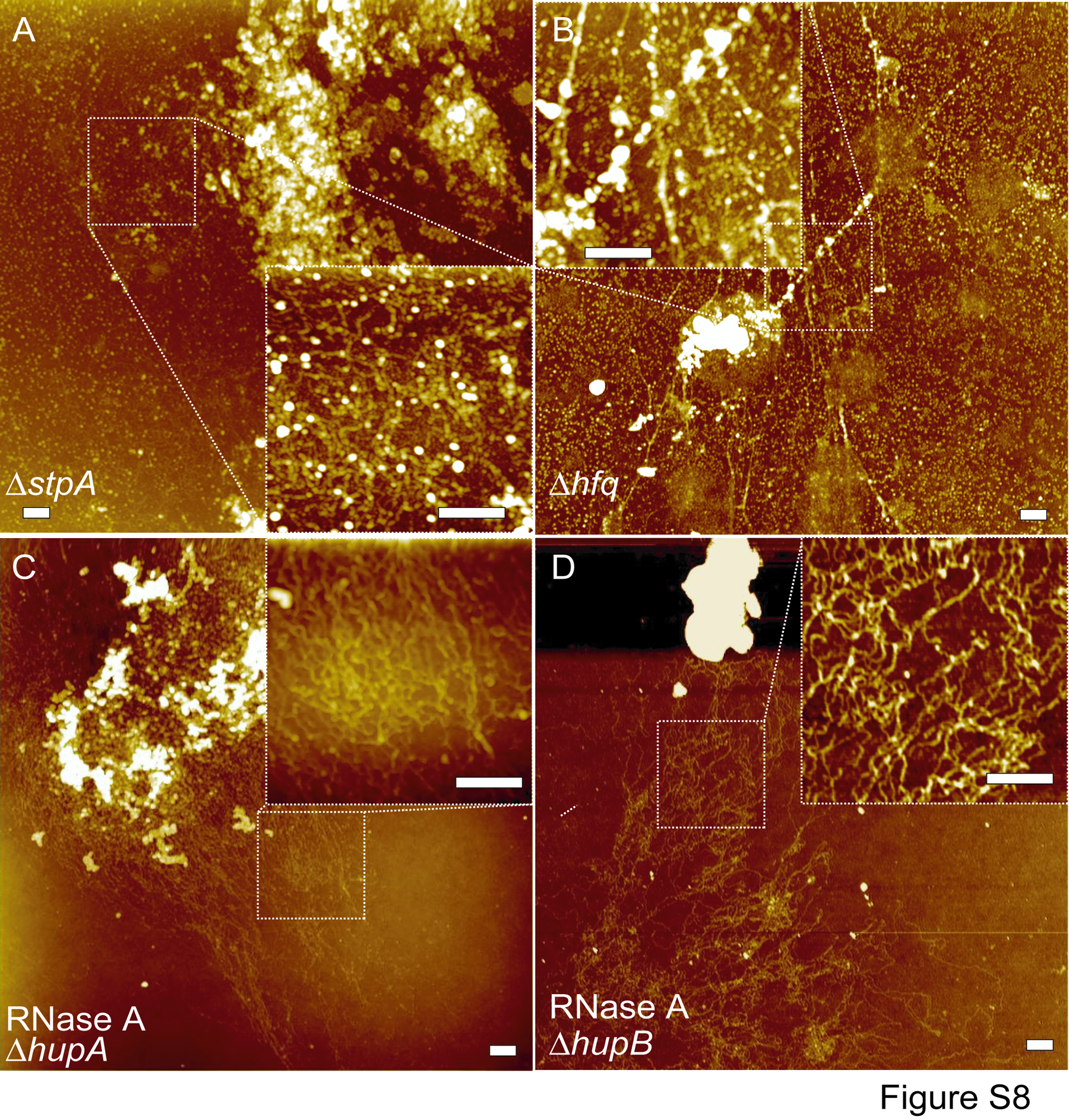

Supplement: Figure S8 — Enlarged AFM images shown in Figures 2I, 2J, 3A and 3B. Lysed log phase E. coli ΔstpA (A), Δhfq (B), ΔhupA treated with RNase A (C) and ΔhupB treated with RNase A (D) cells. (TIF) [file pone.0072954.s008.tif]

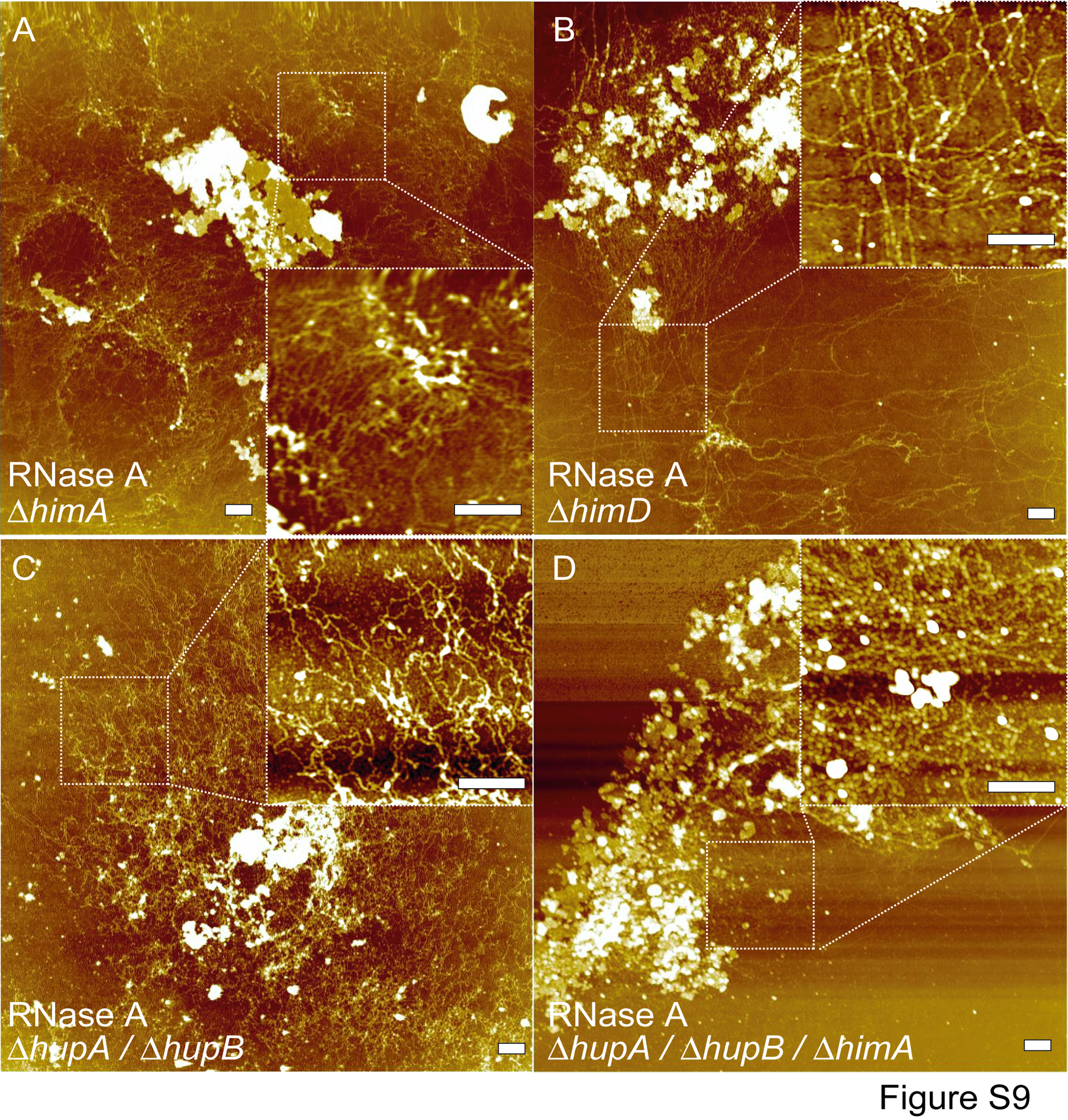

Supplement: Figure S9 — Enlarged AFM images shown in Figures 3C, 3D, 3E and 3F. RNase A treated lysed log phase E. coli ΔhimA (A), Δhim (B), ΔhupA/ΔhupB (C) and ΔhupB/ΔhupB/ΔhimA (D) cells. (TIF) [file pone.0072954.s009.tif]

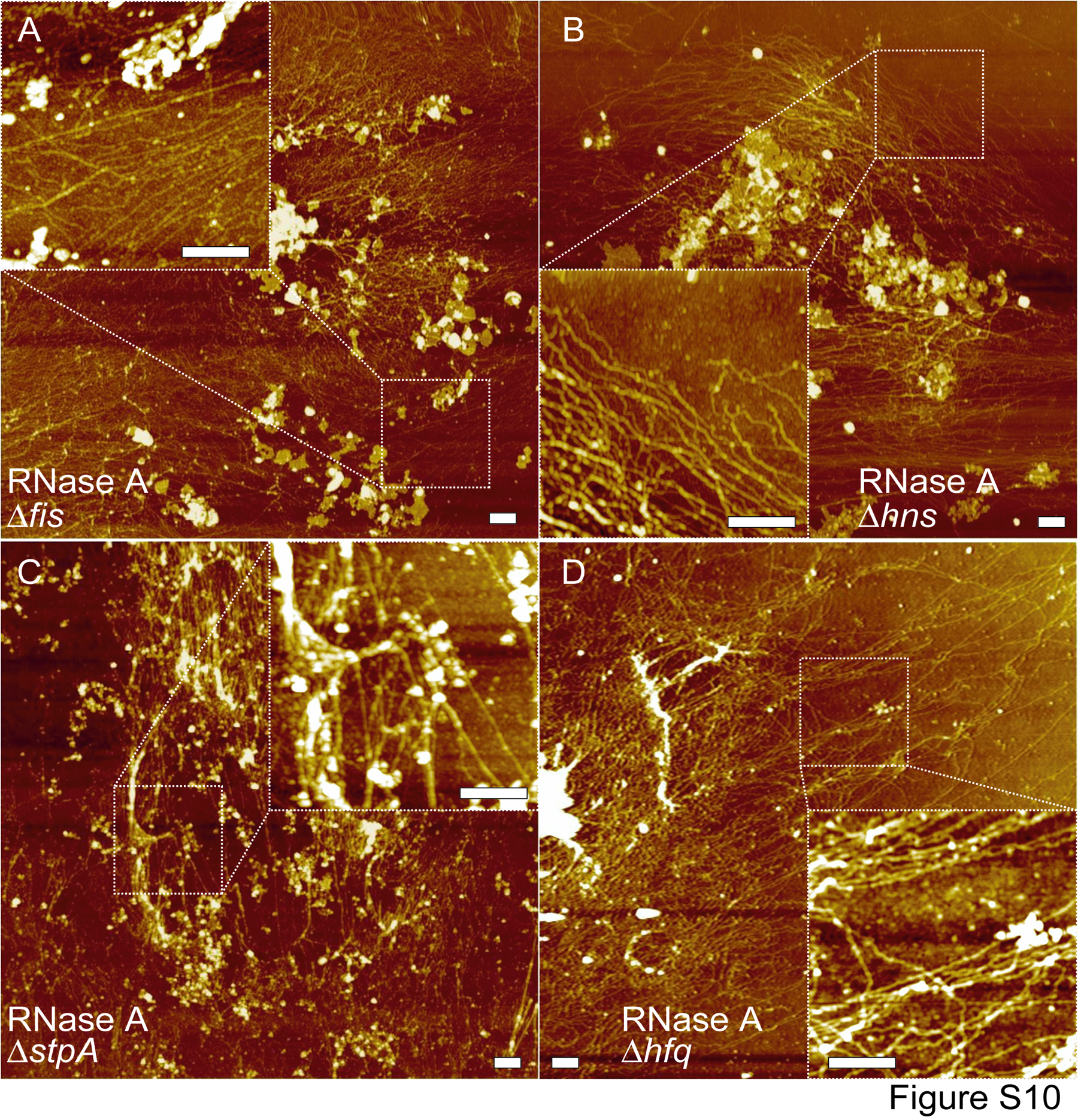

Supplement: Figure S10 — Enlarged AFM images shown in Figures 3G, 3H, 3I and 3J. RNase A treated lysed log phase E. coli Δfis (A), Δhns (B), ΔstpA (C) and Δhfq (D) cells. (TIF) [file pone.0072954.s010.tif]

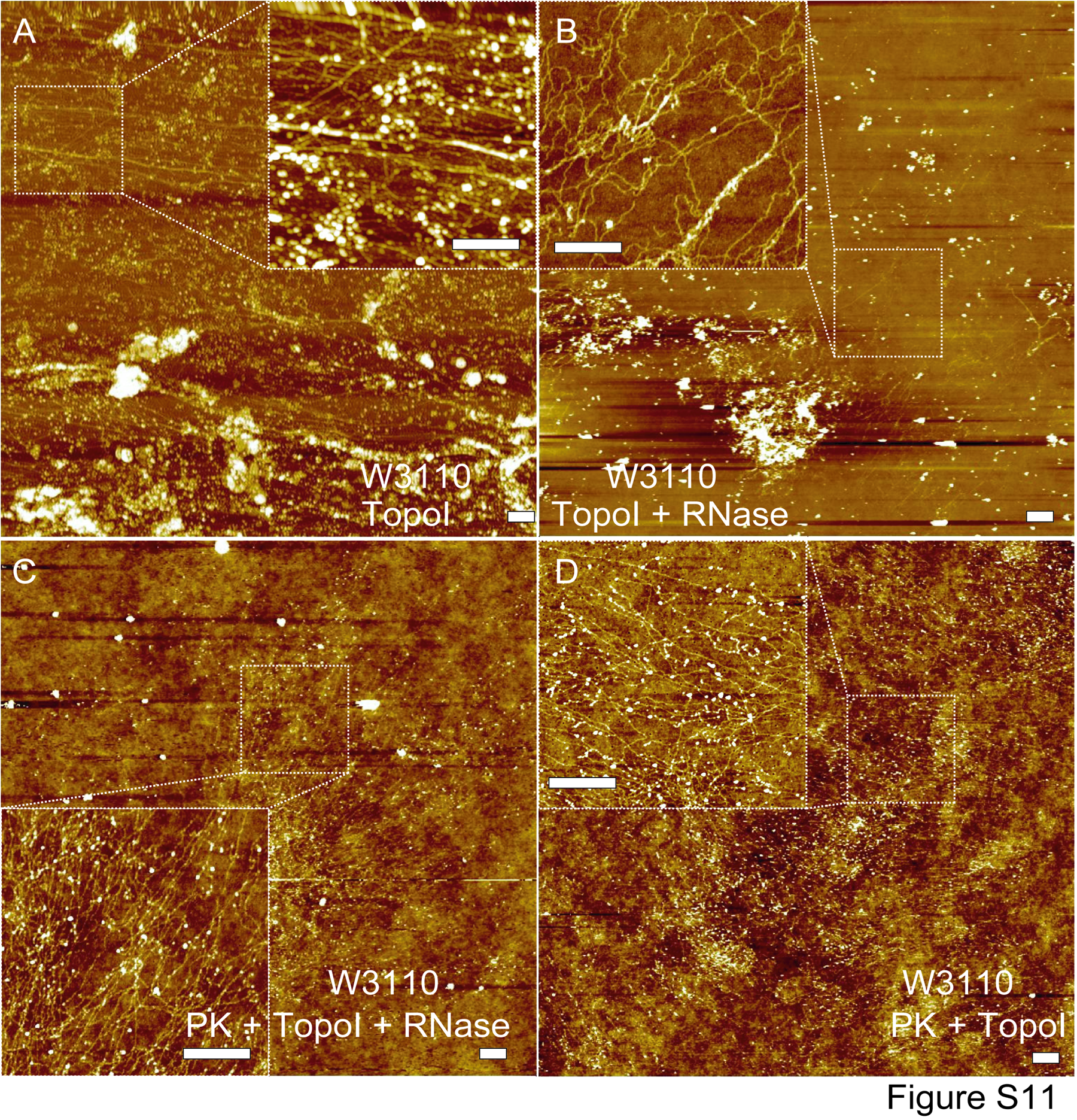

Supplement: Figure S11 — Enlarged AFM images shown in Figures 5A, 5B, 5C and 5D. Lysed E. coli W3110 cells treated with topoisomerase I (A), topoisomerase I and RNase A (B), topoisomerase I, RNase A and protease K (C), and topoisomerase I and protease K (D). (TIF) [file pone.0072954.s011.tif]
